# AI exposure predicts unemployment risk: A new approach to technology-driven job loss

**DOI:** 10.1093/pnasnexus/pgaf107

**Published:** 2025-04-02

**Authors:** Morgan R Frank, Yong-Yeol Ahn, Esteban Moro

**Affiliations:** Department of Informatics and Networked Systems, University of Pittsburgh, Pittsburgh, PA 15216, USA; Digital Economy Lab, Institute for Human-Centered Artificial Intelligence, Stanford University, Stanford, CA 94305, USA; Media Laboratory, Massachusetts Institute of Technology, Cambridge, MA 02139, USA; Connection Science, Massachusetts Institute of Technology, Cambridge, MA 02139, USA; Connection Science, Massachusetts Institute of Technology, Cambridge, MA 02139, USA; Center for Complex Networks and Systems Research, Luddy School of Informatics, Computing, and Engineering, Indiana University, Bloomington, IN 47405, USA; Media Laboratory, Massachusetts Institute of Technology, Cambridge, MA 02139, USA; Connection Science, Massachusetts Institute of Technology, Cambridge, MA 02139, USA; Network Science Institute, Northeastern University, Boston, MA 02115, USA

**Keywords:** artificial intelligence, automation, labor economics, economic resilience, data science

## Abstract

Is AI disrupting jobs and creating unemployment? This question has stirred public concern for job stability and motivated studies assessing occupations’ automation risk. These studies used readily available employment and wage statistics to quantify occupational changes for employed workers. However, they did not directly examine unemployment dynamics primarily due to the lack of data across occupations, geography, and time. Here, we overcome this barrier using monthly occupation-level unemployment data from each US state’s unemployment insurance office from 2010 to 2020 to assess AI exposure models, job separations, and unemployment through a new measure called unemployment risk. We demonstrate that standard employment statistics are inadequate proxies for occupations’ unemployment risk and find that individual AI exposure models are poor predictors of occupations’ unemployment risk states’ total unemployment rates, and states’ total job separation rates. However, an ensemble approach exhibits substantial predictive power, accounting for an additional 18% of variation in unemployment risk across occupations, states, and time compared to a baseline model that controls for education, occupations’ skills, seasonality, and regional effects. These results suggest that competing models may capture different aspects of AI exposure and that automation shapes US unemployment. Our results demonstrate the power of occupation-specific job disruption data and that efforts using only one AI exposure score will misrepresent AI’s impact on the future of work.

**Significance Statement**

AI may disrupt jobs. While numerous studies have explored occupations’ automation risk, they primarily focused on employment which may not fully capture the nuances of job displacement. This study uses a dataset of unemployment insurance claims to quantify “unemployment risk” for various occupations, states, and time. These data challenge the reliability of using employment or wage changes as sole indicators of AI’s impact on jobs. The study reveals that, while individual predictive power is poor, an ensemble approach combining multiple models significantly improves predictions of unemployment risk, job separations, and skill change. This comprehensive assessment highlights the need for diversified labor data when evaluating the impact of AI on the workforce.

## Introduction

AI is one of the most significant modern technological advances. Its wide-ranging impact on our daily lives and jobs has revived fears of technological unemployment among researchers (1–5) and the news media:

- “AI will shrink workforces within five years, say company execs.” CNN (2024 April 5)
- “Almost 65,000 Job Cuts Were Announced In April—And AI Was Blamed For The Most Losses Ever.” Forbes Magazine (2024 May 2)
- “Recent data shows AI job losses are rising, but the numbers don’t tell the full story.” CNBC (2023 December 16)
- “AI could eliminate nearly 8 million jobs in UK, study shows.” ABC News (2024 March 27)
- “AI isn’t coming for your job—at least not yet.” Fortune (2024 May 19)

Accordingly, policymakers strive to address AI’s influence on job stability and the future of work. For example, the 2023 Biden Administration Blueprint for an AI Bill of Rights (6) calls for “training, assessment, and oversight to combat automation bias” and the Executive Order on Safe, Secure, and Trustworthy AI highlights the “promise of improved productivity but also the dangers of increased workplace surveillance, bias, and job displacement.” Many existing studies estimate occupations’ automation or exposure risk from AI (7–13), so which of these scores should policy makers use to focus resources to the workers facing job disruption? Policymakers, employers, and workers need to know where, when, and for which occupations AI exposure (2, 14–17) predicts job loss, unemployment, or the need for workers to reskill.

The disconnect between public concern and research practices has created a critical knowledge gap. Researchers have primarily concentrated on occupations’ employment and wages because of readily available data from the US Bureau of Labor Statistics (BLS) and the prominence of the skill-biased technological change theory (SBTC) (18). This theory suggests that technology can enhance worker productivity, potentially increasing labor demand depending on market conditions (i.e. demand elasticity). However, applications of these exposure estimates by government and industry institutions including the Brookings Institution (19), the BLS (20), and the Organisation for Economic Cooperation and Development (OECD) (21–24) have not observed a significant decrease in employment for occupations deemed “high-exposure.” Rather than concluding that AI does not disrupt jobs, the US BLS (20, 25, 26) indicates that new data are required to fully understand AI-driven job loss.

We argue resolving these mixed results requires a specific investigation into AI exposure and job loss, in addition to existing studies of employment and wages. Research has focused on employment or wages for occupations because SBTC suggests that technology contributes to wage inequality, employment growth for skilled workers, and employment loss for unskilled workers. Other reasons include the fact that these statistics are readily available from the BLS and that workers will mostly not lose jobs but instead adapt to technology-driven shifts in an occupation’s required workplace activities (i.e. within-occupation skill change). However, workers who do not adapt can experience job separations (i.e. quits or fires) (27–29) and then unemployment if they fail to find their next job quickly. It is not possible to quantify these dynamics from employment or wages because—as we show—unemployment or job separations can increase or decrease even as employment grows (20, 22). For instance, new workers may replace other workers if they can complement new technology, thus boosting employment and/or wages for the exposed occupation even though a disruption occurred (e.g. bank tellers and ATMS (30, 31)). This means that if occupations with reduced demand also produce unemployment, then this unemployment corresponds to industrial reallocation (e.g. because of AI) and measures of workers’ probability of becoming unemployed would reveal these heterogeneous effects across workers, industries, and regions.

In this article, we address this challenge by answering the following research questions:

RQ1: How can we quantify the likelihood of unemployment across US states and sectors? To solve this problem, we build a high-resolution dataset that documents monthly unemployment counts by occupation from each US state’s unemployment insurance office and use this data to calculate a worker’s probability of receiving unemployment benefits (called *unemployment risk*) based on their occupation, state, and time period. We show that changes to an occupation’s employment share or to total unemployment in a state do not predict unemployment risk.
RQ2: With at least a decade of studies modeling occupations’ exposure to AI, which scores predict occupations’ unemployment risk, job separations, or skill change? Is AI a potential factor in job loss? We explore existing AI exposure models and test which scores, if any, predict occupations’ unemployment risk, job separations (i.e. job quitting or firing), or within-occupation skill change using multiple regression analyses. We find that individual AI exposure studies are not predictive of unemployment risk, job separations, or skill change. However, an ensemble model combining approaches from these individual studies is predictive even after controlling for regional fixed effects, temporal effects (e.g. seasonality), and occupations’ skill requirements.
RQ3: For which states or economic sectors do AI exposure models predict unemployment by occupation, job separations, or skill change? We analyze which individual AI exposure models are most applicable in different parts of the US economy (e.g. by state or occupation) and observe strong geographical heterogeneity in the applicability of each AI exposure score.

Our results suggest that predicting AI job loss or unemployment cannot rely on any one score. Combined, these results demonstrate that employment and wage data may miss other detrimental labor dynamics from AI technology and that efforts using only one AI exposure score will misrepresent AI’s impact on the future of work.

## Quantifying occupations’ unemployment risk

Research has concentrated on occupations’ employment or wage statistics because workers can adapt to technology-driven changes in job activities without job loss. The US BLS Occupation Employment and Wage Statistics (OEWS) describes occupations’ employment and wages while the prevailing SBTC theory highlights that technological advancements can enhance worker productivity, potentially increasing demand for labor depending on market conditions and demand elasticity. Yet, at the state level, job loss dynamics experienced by different workers are obfuscated in the unemployment and job separation statistics from the BLS Local Area Unemployment Statistics (LAUS) or Job Openings and Labor Turnover Survey (JOLTS) which only report total counts or rates unstratified by sector or occupation. Here, we quantify job loss across US states, occupations, and time (i.e. months) using data from each state’s unemployment benefits office detailing the number of unemployment benefit recipients by state, month, and most-recent occupation; these data reflect the entire US population of continued unemployment claimants and the total number of unemployment recipients from this data is highly correlated with more typical monthly total unemployment from BLS LAUS (see Supplementary material Table S1). Combined with employment statistics, we calculate an occupation’s *unemployment risk* according to


(1) $$p(unemp\;|\;soc,s,t)=\frac{n(unemp\;|\;soc,s,t)}{n(unemp\;|\;soc,s,t)+n(emp\;|\;soc,s,t)}$$

where *emp* indicates a worker is employed, *unemp* indicates a worker is unemployed, *soc* denotes an occupation’s two-digit Standard Occupation Classification (SOC) code, *s* denotes a state, *t* denotes a time period (i.e. year and month), and *n* captures the number of employed or unemployed workers by occupation, state, and time period. n(unemp|soc,s,t) comes from the monthly detailed unemployment data while n(emp|soc,s,t) comes from annual BLS OEWS (see Supplementary material Fig. S1). Given a state and time, p(unemp|soc,s,t) is the probability that a worker with occupation *soc* receives unemployment benefits; note that p(unemp|soc,s,t) is relative to the labor force and not relative to total employment or total unemployment. Unemployment risk varies by occupation (e.g. construction workers versus transportation workers) while controlling for states’ total labor market size and occupations’ local employment share (e.g. more retail workers may receive unemployment benefits in a state where retail workers are a larger share of employment).

Many studies focus on an occupation’s change in employment following a technological disruption, so one might assume that an occupation’s rising employment would directly correspond to falling unemployment risk. Similarly, a more typical analysis of a state’s total unemployment rate may sufficiently explain variation in unemployment risk as being largely driven by systemic economic factors. Using Bayes’ Theorem, we can relate an occupation’s unemployment risk to its local employment share and the state’s total unemployment rate according to


(2) $$p(unemp\;|\;soc,s,t)=\frac{\;p(soc\;|\;unemp,s,t)\cdot p(unemp\;|\;s,t)}{p(soc\;|\;emp\cup unemp,s,t)},$$

where p(unemp|s,t) is the total unemployment rate from BLS LAUS and p(soc|emp∪unemp,s,t) is the share of the local labor force associated with *soc*.

**Fig. 1.**
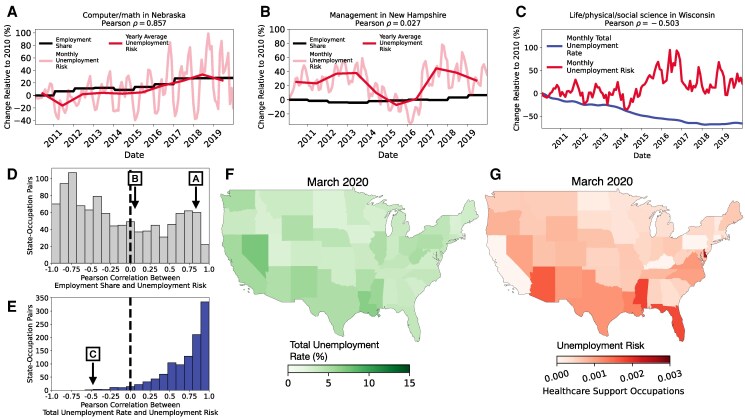
Total unemployment and occupations’ employment share are not proxies for an occupation’s unemployment risk. A) Relative change in employment and unemployment risk for Computer/Math occupations in Nebraska rise together over time. B) Relative change in employment and unemployment risk for Management occupations in New Hampshire are not significantly correlated over time. C) The relative change in the risk of unemployment in the life, physical, and social sciences occupations of Wisconsin is negatively correlated with total unemployment over time. D) Across all occupation–state pairs, an occupation’s employment share has no predictable relationship to its unemployment risk over time. E) Across all occupation–state pairs, a state’s total unemployment is typically correlated with occupation’s unemployment risk over time, but not always. A–E) are based on data from January 2010 through December 2019. F) States’ total unemployment rate in March 2020. G) Unemployment risk by state for Healthcare Support occupations in March 2020.

Is an occupation’s unemployment risk just the opposite of its employment over time? In general, an occupation’s employment share over time within a state has no consistent empirical relationship with the occupation’s unemployment risk (see Fig. 1D).^a^ In general across all occupations and states, employment share has a Pearson Correlation with unemployment risk of ρ=−0.13 ($P\text{-value}<10^{-5}$) but many occupation–state pairs deviate from this aggregate result. For example, unemployment risk for Computer and Math occupations in Nebraska is positively correlated with employment share over time (Pearson ρ=0.86. See Fig. 1A) and uncorrelated for Management occupations in New Hampshire (Pearson ρ=0.03. See Fig. 1B). Thus, an occupation’s employment share is not a reliable proxy for its unemployment risk.

Is an occupation’s unemployment risk driven mostly by a state’s total unemployment? In general, across all occupations and states, the total unemployment rate in the state has a Pearson correlation with unemployment risk of ρ=0.19 (*P*-value$<10^{-5}$). Mostly, an occupation–state pair’s unemployment risk covaries with the state’s total unemployment rate over time but not always (see Fig. 1E).^b^ For example, Wisconsin’s unemployment risk for Life, Physical, and Social Science occupations is significantly negatively correlated with total unemployment rate over time (see Fig. 1C). Similarly, a cross-sectional mapping of an occupation’s unemployment risk across states identifies where particular workers are experiencing increased likelihood of receiving unemployment benefits. For example, in March 2020, during the rise of the 2020 US COVID Recession, states with the highest total unemployment rates (see Fig. 1F) were not the same states with Healthcare Support occupations experienced the highest unemployment risk (see Fig. 1G).

We provide an analogous analysis of wage shifts versus unemployment risk for occupation pairs over time (see Supplementary material Fig. S9). While it’s tempting to assume that increases in earnings will correspond to decreased unemployment risk, empirically, this is not always the case. For example, according to BLS OEWS, Art and Entertainment occupations in Vermont and Life, Physical, and Social Science occupations in New Mexico have both increasing average annual wages and increasing unemployment risk. Thus, as with employment shifts over time, wage shifts are a poor proxy for occupations’ unemployment risk.

## A decade of estimating exposure to AI

We use unemployment risk to test the predictive power of existing models of AI exposure, some of which have been used in policymaking (e.g. the OECD (22, 24)). Models for occupations’ exposure to AI and/or robotics have emerged over the last decade while emphasizing different technologies and industries. For example, recent studies focus on generative AI (32) or large language models (LLMs) (13). But, it remains unclear which labor markets (e.g. states) or occupations are best predicted by each approach, and how to combine their relative strengths. This observation calls into question predictions of labor market outcomes based on collections of AI exposure scores (e.g. regressing industry labor share compared to exposure scores to estimate automation-driven declines in labor share (33)).

Here, we will focus on AI exposure while leaving exposure to robotics mostly as future work. Motivated by the SBTC theory (18, 34, 35), a first wave of studies argued that college-educated, high-skill workers who perform cognitive tasks are complemented by technology while low-skill workers who perform manual tasks are more frequently substituted by technologies like robotics (36, 37). In these studies, workers are mostly identified by their education or their industry. A second wave of studies used BLS O*NET data to model occupations based on their skill requirements (7, 8). Most recently, a third wave of studies compares technological capabilities to job descriptions (i.e. a task-based approach (33)) by surveying machine learning (ML) experts (9), surveying gig workers (10), applying natural language processing to technology patents (11), or even asking the AI to self-assess (13). Table 1 summarizes these studies across all three waves (see Supplementary material Section 1 for more information). These studies span the previous decade and, while theory and methodology vary, none of them provide causal evidence of the role of technology in labor outcomes; in particular, none say anything directly about job loss except as a motivation for the study. In fact, almost all studies explicitly state that the relationship between their scores and job loss is uncertain (e.g. “We make no attempt to estimate the number of jobs that will actually be automated and focus on potential job automatability over some unspecified number of years. (7)”). Instead, these studies argue that their measures are useful because they correspond to decreasing employment or wages or to occupations with lower education requirements (except for Ref. (13) which suggests that higher education work is more exposed).

**Table 1.** Waves of studies estimating AI exposure by occupation.

Wave	Study	Year first available	Scores	Description
1	O*NET Bachelors	2003	(denoted **%college**)	The fraction of workers in an occupation with a bachelor’s degree.
	Acemoglu and Autor (18)	2011	Computer usage (**Comp.Use**), Routine cognitive (**R.Cog.**), Routine manual (**R.Man.**)	Assess occupations on computer usage, routineness, and cognitive or manual requirements.
2	Frey and Osborne (7)	Preprint 2013	Probability of computerization (**auto**)	Combined a subset of occupation skills with subjective assessments of fully automatable or nonautomatable occupations.
	Arntz et al. (8)	2016	Probability of computerization (**auto2**)	Considered a complete set of occupations’ skills to assess automation risk in OECD countries.
	O*NET degree of automation	2016	(**Deg.Auto.**)	The relative amounts of routine versus challenging work the worker will perform as part of a job.
3	Brynjolfsson et al. (9)	2018	Suitability for machine learning (**SML**)	Surveyed ML experts in order to assess occupations’ task suitability for ML.
	Felten et al. (10)	2018	(**AI2**)	Crowdsource gig workers to establish connections between AI application capabilities and occupation abilities.
	Webb (11)	2019	% AI exposure (**AI**), % Software Exposure (**Software**), % robot exposure (**Robot**)	Uses NLP to compare technology patents to occupation tasks.
	Eloundou et al. (13)	2024	Exposure to LLMs (**LLM**)	Authors and annotators with exposure to LLMs performed assessments and the AI self-assessed workplace activities that LLMs could perform.

Methodologies have evolved from solely theoretical motivations (wave 1) to greater specificity into occupations’ skills (wave 2) to connecting skills to the capabilities of specific technologies (wave 3). Scores are taken from each study; short-hands for each score are provided in parentheses and bold print.

**Fig. 2.**
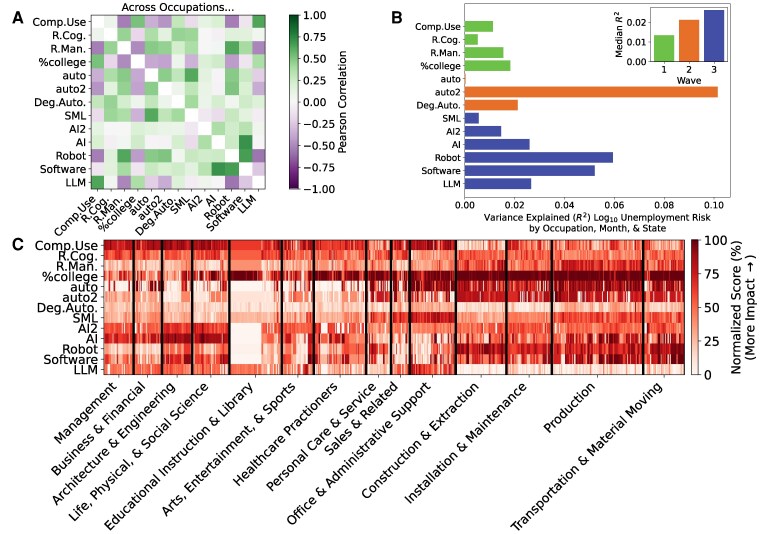
Technological exposure scores are not consistent with each other and cannot individually predict unemployment risk well. A) The Pearson correlation of pairs of AI exposure scores across occupations. Scores are ordered according to the study’s wave (see Table 1). B) The variation in unemployment risk explained by each AI exposure score. Colors indicate the score’s wave. The inset reports the median variance explained ($R^{2}$) for scores by wave. C) A heat map detailing occupations’ AI exposure scores (color). Occupations are grouped according to their major occupation (*x*-axis).

Since each study shares the common goal of modeling AI exposure, we expect that their estimates will mostly agree with each other. However, we find that exposure scores across studies are not strongly correlated (see Fig. 2A) and can even be anti-correlated when compared across occupations. For example, Webb’s measure for Software usage is negatively correlated with Acemoglu and Autor’s measure of Computer usage and negatively correlated with the national fraction of workers with a bachelor’s degree. The strongest correlation among individual scores is between AI exposure and Software exposure scores from the same study (11) ($R^{2}=0.494$), but the variance explained from one score to the next is typically small (average $R^{2}=0.111$ and median $R^{2}=0.068$). This disagreement among exposure scores persists across individual occupations and is present across all occupation categories (see Fig. 2C). This lack of correspondence may be due to different methodologies, differences in the data available, and differences in the technology focused on in the study (e.g. “computerization (7)” versus supervised machine learning (9) or LLMs (13)). Hence, it is important to assess the applicability of each study’s scores to specific geographies and sectors before solely relying on one study’s findings to determine the impact of AI on workers.

## Exposure estimates and unemployment risk

Do any AI exposure scores predict unemployment risk or other job disruption statistics? If so, then technology may be detrimental to some US workers, and comparing different AI exposure scores will reveal a clear winner that policymakers and workers can use to predict severe job disruption due to automation. But, if not, then several possible scenarios arise. First, it may be that technology is not a major factor shaping a worker’s likelihood of receiving unemployment. In this case, no reasonable effort to measure AI exposure would predict unemployment risk or job separations. Second, if technology indeed impacts workers, then the disagreement among exposure scores (see Fig. 2A and C) may indicate that researchers have yet to converge on good measures for AI exposure thus highlighting the need for new research into predicting occupations’ exposure to automation from technology. Third, if each exposure score describes different types of AI exposure or performs well in different geographies or sectors, then individual scores might predict only small shares of unemployment risk individually but combine to explain a larger share.

Our analysis supports the last scenario. First, individual exposure scores do not strongly predict unemployment risk (see Fig. 2B). For example, occupations’ Computer Usage (18) explains <2% of the variation in unemployment risk ($R^{2}=0.013$) across occupations, states, and time. The national share of an occupation’s workers with a bachelor’s degree yields $R^{2}=0.018$. The most predictive model comes from Arntz et al. (8) ($R^{2}=0.107$), which was a response to another wave 2 study from Frey and Osborne (7) ($R^{2}=0.001$). On aggregate, the median variance explained increased across the three waves of exposure scores (see Fig. 2B inset) from $R^{2}=0.015$ for wave 1 to $R^{2}=0.027$ for wave 3. The most predictive score explains only 10.7% of variation in unemployment risk across occupations, states, and months.

Yet, taken together as an ensemble, AI exposure scores predict much more variance in unemployment risk across occupations, states, and time. We combine all of the exposures scores (see Table 1) as variables in a LASSO regression model to form an ensemble model where the LASSO training algorithm can weight each score using a regression coefficient and even ignore certain exposure scores by applying a coefficient of zero. The LASSO regression model combining all exposure scores accounts for 29.8% of the variation in unemployment risk (see Table 2 model 1). As in the other AI exposure studies referenced here, while predictive, this simple approach misses several potential confounds which limits the causality that can be inferred. For example, a worker’s skills or education may determine both their AI exposure and employers’ likelihood to hire them. Or, automation may vary by state and/or time depending on the local economy’s industrial composition. For example, production line robotics would impact blue-collar rural economies more than white-collar urban economies (38).

**Table 2.** Combining AI exposure scores from all studies substantially improves predictions of unemployment risk.

Dependent variable: Log_10_ unemployment risk by occupation, month, and state			
Variable	Model 1	Model 2	Model 3
Computer usage (18)	0.771***		0.493***
Routine cognitive (18)	− 0.224***		− 0.192***
Routine manual (18)	− 0.116***		0.000
Probability of computerization (7)	− 0.009		− 0.314***
Probability of automation (8)	0.702***		0.364***
Degree of automation	0.001		0.000
Suitability for machine learning (9)	− 0.117***		− 0.152***
AI exposure (10)	− 0.109***		0.000
% AI (11)	− 0.000		0.055***
% Robot (11)	0.366***		0.518***
% Software (11)	− 0.000		− 0.000
LLM (13)	− 0.161***		− 0.044***
% Workers w/ Bachelors		0.039***	− 0.018***
O*NET PCA	No	Yes	Yes
Year F.E.	No	Yes	Yes
Month F.E.	No	Yes	Yes
State F.E.	No	Yes	Yes
$R^{2}$	0.298	0.574	0.755
adj. $R^{2}$	0.298	0.574	0.755
$\$\$P_{\mathrm{val}}<0.1^{*}\$\$,\$\$P_{\mathrm{val}}<0.01^{**}\$\$,\$\$P_{\mathrm{val}}<0.001^{***}\$\$$			

$P_{\mathrm{val}}<0.1^{*}$ , $P_{\mathrm{val}}<0.01^{**}$, $P_{\mathrm{val}}<0.001^{***}$. Combined into a single linear model, AI exposure scores capture 29.8% of the variation in unemployment risk (model 1). Compared to a baseline model with controls for year, seasonality, state, occupations’ educational requirements and occupations’ O*NET skill requirements (model 2), the combined model accounts for 75.5% variation in unemployment risk (model 3). All variables were centered and standardized before LASSO regression. Unemployment risk is calculated using monthly data on unemployment recipients from each US state’s unemployment insurance office (N=140,274).

**Fig. 3.**
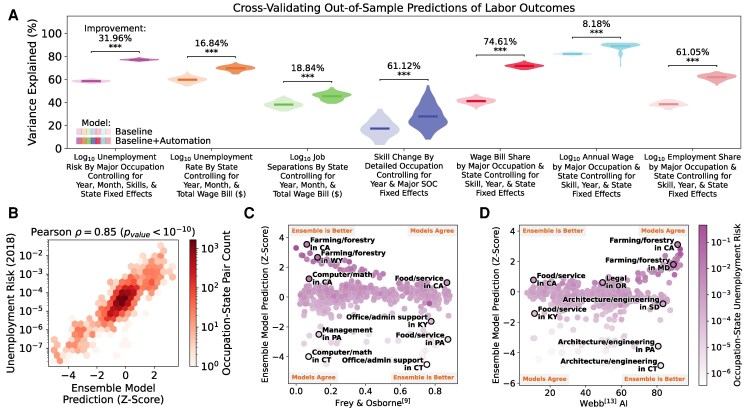
Combining AI exposure scores from all studies improves predictions of labor outcomes and reveals shortcomings in individual scores. A) Comparing out-of-sample variance explained with and without AI exposure scores. Each analysis includes ten independent trials of 10-fold cross-validation training a LASSO regression model; this produces a distribution of 100 observations of each model’s predictive performance. Horizontal lines represent the average of each distribution. For each labor outcome, including the combined AI exposure model significantly improves predictive performance (i.e. two-sample t-test *P*-value $<10^{-3}$). The percentages indicate the average factor improvement in variance explained for each labor outcome when the combined AI exposure model is added to the baseline model. B) Ensemble model’s predictions for unemployment risk by occupation and state in 2018 are strongly correlated to actual unemployment risk. Monthly unemployment data were averaged to produce annual estimates which were used to calculate unemployment risk. C) Compared to the ensemble model (y-axis), predictions from Frey and Osborne’s (7) (*x*-axis) underestimate unemployment risk (color) for Computer/math occupations in California and overestimate unemployment risk for Maintenance occupations in Connecticut using 2013 data. D) Compared to the ensemble model, predictions using Webb’s AI score (11) underestimate unemployment risk for Food prep/service occupations in California and overestimate unemployment risk for Architecture/engineering occupations in South Dakota using 2019 data.

While other potential confounds may exist, we expect that many of these factors would affect the correlation between technology and unemployment through skills or education. For example, nearly all of the association between exposure scores—including occupations’ educational requirements—and occupations’ annual wage is confounded by occupations’ skill requirements (see Fig. 3A which is explained below and Supplementary material Tables S10–S12). This observation informs our baseline model, which controls for occupations’ O*NET skill requirements (i.e. using principal component analysis. See Supplementary material Fig. S2), the national fraction of workers in each occupation with a bachelor’s degree, and fixed effects for year, month, and state. State fixed effects control for regional variables (e.g. access to a port or natural resources), year fixed effects control for long-term temporal dynamics (e.g. recovering from the Great Recession), and monthly fixed effects control for seasonal trends (e.g. for most occupation–state pairs, unemployment risk peaks during summer months. See Fig. 1A and B). This baseline model accounts for 57.4% of variation in unemployment risk across occupations.

Adding AI exposure scores to the baseline model accounts for an additional 18.1 percentage points of variation in unemployment risk (i.e. 75.5% variation explained. See Table 2 models 2 and 3). We observe similar performance gains using out-of-sample cross-validation (see Fig. 3A). We provide full regression tables in Supplement material Tables S2 and S3. A large proportion of variation explained indicates that many sources of unemployment risk affect workers through education, workers’ skills, seasonality, and regional factors, but the additional 18.1% of variation explained with the inclusion of the ensemble model (i.e. model 3) suggests substantial influence from technology. Since most potential confounds—although not all—are controlled for in the baseline model, we interpret these results as evidence that indeed technology contributes to workers’ unemployment risk.

How do the different AI exposure scores contribute to the ensemble model? The ensemble model is differentially leveraging the strengths of each model to produce better predictions (e.g. see Fig. 3B). Comparing individual scores to the ensemble model’s predictions highlights the states and occupations where that methodology under performs. For example, predictions from Frey and Osborne’s (7) underestimate unemployment risk for Computer/math occupations in California and overestimate unemployment risk for Office/Administrative occupations in Connecticut in 2013 (i.e. the year that scores became available. See Fig. 3C). As another example, predictions using Webb’s AI score (11) underestimate unemployment risk for Food prep/service occupations in California and overestimate unemployment risk for Architecture/engineering occupations in Pennsylvania using 2019 data compared to the predictions of the ensemble model (see Fig. 3D). In Supplementary material Figures S3–S7, we provide Shapley values for model 1 and again find that most exposure scores contribute to the model’s overall predictive performance.

Since many AI exposure studies compare their scores to occupations’ employment and wages, we similarly predict occupations’ per state annual wage bill share, employment share, and annual wages according to the BLS (see Fig. 3A). Including the exposure scores improves the predictions of wage bill share by 74.61% over the baseline model which controls for occupations’ skill requirements, state, and year. Further analysis shows that this predictive performance is primarily due to improvements in employment share predictions when AI exposure scores are included in the model. Predictions of occupations’ average annual wage by state and year is only slightly improved by 8.18% with the inclusion of exposure scores when occupations’ skill requirements are included in the baseline model. We provide full regression tables for these analyses in Supplementary material Tables S10–S13.

## Technology, job separations, and changing skill demands

**Fig. 4.**
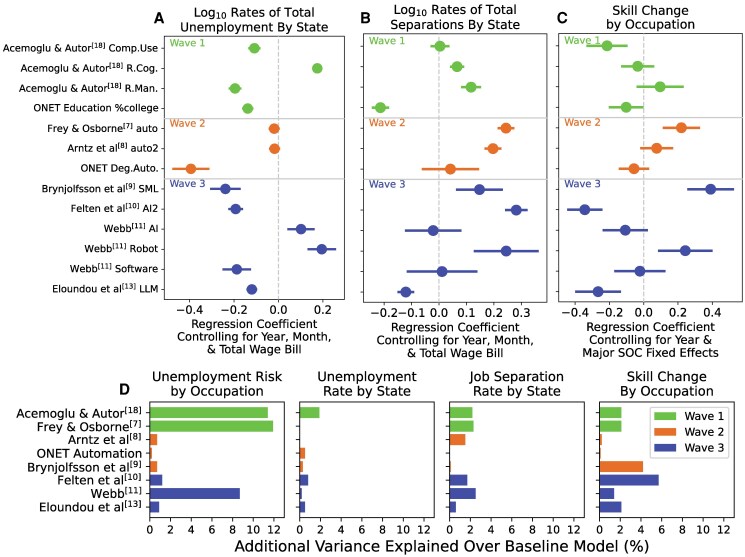
AI exposure scores individually predict small amounts of variation states’ total unemployment, states’ total job separations, and within-occupation skill change. Linear regression coefficients reporting the relationship between each exposure scores and A) total unemployment rate, B) total job separation rates, and C) within-occupation skill change. We provide 95% CI. Colors represent study waves. D) For each labor outcome, the additional predictive performance (i.e. adjusted $R^{2}$) when exposure scores are added to the baseline model. In all plots, all variables were centered and standardized. Full regression tables are available in the Supplementary material.

Unemployment is an extreme result of job disruption, so what about other labor outcomes? For example, job separations can occur without contributing to unemployment if displaced workers quickly find new employment (39). Similar to unemployment risk, depending on the score used, correlations varied between states’ AI exposure (see Supplementary material Section 2) and states’ job separation or total unemployment rate (see Fig. 4A and B, respectively). On aggregate, AI exposure from Felten et al. (10) was most positively associated with job separation rates while Webb’s (11) Robot exposure was most positively associated with unemployment rates (see Fig. 4D). However, each individual score contributed only a small amount of variance explained compared to the baseline model. Yet, combining all scores into a single model yields a 16.8% improvement in explained total unemployment rate variation by state and month and a 18.8% improvement for job separation rates by state and month according to out-of-sample cross-validation (see Fig. 3). Full regression tables are provided in Supplementary material Tables S4–S7.

Does technology shape labor outcomes by changing skill demands? Technology rarely automates entire occupations wholesale but instead automates specific workplace activities (13, 14, 18). These changes may be subtle without producing job separations or unemployment if workers adapt. Some studies hypothesize that AI (11, 40) or machine learning (ML) (9) would change skill demands within occupations slowly enough for workers to adapt their skills and/or find new employment. However, reskilling or up-skilling still imposes an adjustment cost on both workers and employers (41, 42).

We use occupation skill profiles from the BLS O*NET database to track changes to skill demands within about 700 different occupations (see Supplementary material Fig. S16 and Tables S8 and S9). Routine Manual work was the only wave 1 AI exposure score positively associated with skill change (see Fig. 4C). From wave 2, both Frey and Osborne’s (7) and Arntz et al.’s (8) scores were positively associated with skill change. Technology-specific exposure scores from wave 3 yielded mixed results. Suitability for ML (9) predicts greater skill change, while AI exposure from Felten et al. (10) predicts less change. This is especially surprising because these studies employed similar methodologies; both studies surveyed people, either from CrowdFlower or from computer science PhD students, about the specific capabilities of AI or ML to produce their occupation scores. Ultimately, AI exposure according to Felten et al. (10) yielded the largest gain in predictive performance, adding 5.7% variance explained (i.e. adjusted $R^{2}$) compared to the baseline fixed effects model (see Fig. 4D). However, adding the combined AI exposure model to a baseline model controlling for year and occupation fixed effects produces a 61.1% improvement in variation explained (see Fig. 3). Summary skill change statistics and full regression tables are provided in Supplementary material Tables S8 and S9.

## Where and when to use each automation exposure score

**Fig. 5.**
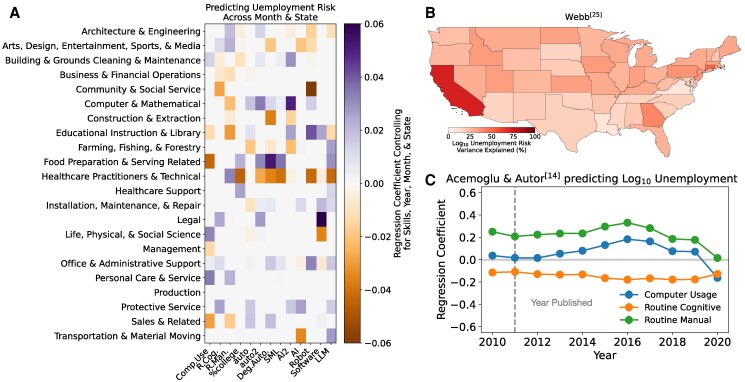
The effectiveness of AI exposure scores varies by occupation, year, and state. A) AI exposure scores’ correspondence with unemployment risk across states and months. Individual regressions were run for each pair of major occupation and AI exposure scores while controlling for skills, month, year, and state. We report regression coefficient estimates with *P*-value $<10^{-2}$. B) The performance of scores from Webb (11) in combination with year and month fixed effects for predicting unemployment risk by occupation. Individual regressions were performed for each state. C) For each year, we regress scores from Acemoglu and Autor (18) against unemployment risk by occupation with state and month-fixed effects. We provide 95% CI for coefficient estimates. In all plots, all variables were centered and standardized before analysis. Similar plots for other AI exposure scores are provided in the Supplementary material.

Examining AI exposure scores’ performance across occupations, locations, and time periods reveals how each score contributes to a combined model. For example, unemployment risk for Sales workers across states was negatively associated with the Routine Cognitive score (18) and positively associated with Suitability for ML (9) (see Fig. 5A. Analysis included year and month fixed effects). As an example of heterogeneous spatial performance, combined with year and month fixed effects, % AI, Robotics, and Software scores from Webb (11) are most predictive of unemployment risk by occupation in California compared to other US states (see Fig. 5B). However, other AI exposure scores yielded more even performance across states. For example, state-level scores derived from Frey and Osborne (7) yielded roughly equal predictive performance across states when predicting total job separation rates (see Supplementary material Section 8 for similar maps of other exposure scores). Similarly, the performance of individual exposure scores can vary over time. For example, Computer Usage (18) was increasingly predictive of unemployment risk before 2016 but became less strongly associated with risk thereafter—even becoming negatively associated with unemployment risk in 2020 perhaps due to more work-from-home activities during the COVID pandemic (see Fig. 5C). As another example, using scores from Webb (11), percentage exposure to Robots indicated greater total job separation rates in states except for the year 2017 when exposure to Software became positively associated with separations (see Supplementary material Figs. S13–S15). Thus, the best model for predicting labor outcomes may vary depending on the spatial or industrial context.

## Discussion

As workers and policy makers shape the future of work, they must model potential job loss from AI. Existing automation studies acknowledge this fact and almost universally motivate their work with the potential for AI to create job loss. However, lacking data on job loss by occupation, these studies instead compare their automation scores with occupations’ employment or wage changes. This analysis is valuable and may even describe a majority of the labor outcomes from technological change as workers gradually adapt their skills to occupations’ changing skill demands. But, armed with data from each US state’s unemployment insurance office, we show that an occupation’s unemployment risk has no consistent relationship with its employment changes over time (see Fig. 1A, B, and D). Similarly, occupations’ wage dynamics over time offer no consistent relationship with unemployment risk (see Supplementary material Figs. S8 and S9). Therefore, analyzing employment and wage shifts alone (e.g. looking for decreasing employment or wages) will miss the workers who are experiencing job loss and unemployment (i.e. for any reason, not just technology).

The Skill-Biased Technological Change (SBTC) theory posits that technological advancements disproportionately benefit skilled workers over unskilled workers. This results in an increase in demand for skilled labor and, consequently, an increase in wage inequality between skilled and unskilled workers. Accordingly, AI exposure studies (9, 11, 14, 18, 20, 40) claim that within-occupation skill change is the most likely outcome from new technology thus motivating their analysis of occupations’ exposure scores and employment or wage shifts. While this approach may capture the symptoms of within-occupation skill change, it does not quantify skill change directly and may miss occupations that experience subtle skill change. Instead, our study leverages over a decade of the annually updated O*NET database from the US BLS (although each occupation is updated only every 5 years) to find that AI exposure scores significantly improve predictions of within occupation skill change (i.e. a 61.1% factor improvement. See Fig. 3).

As federal and state policies prepare for new technology and the future of work (e.g. Illinois Future of Work Act 2021, 2021 IL H 645, and the National Artificial Intelligence Initiative Act of 2020 [NAIIA]), how should government and policy analysts quantify AI exposure? More than a decade of research has produced multiple efforts estimating occupations’ AI exposure and these exposure scores have been used in government and industry analyses, including by the Brookings Institution (19), the US Bureau of Labor Statistics (20), and the Organisation for Economic Cooperation and Development (OECD) (21–24). The lack of agreement among different scores and their poor individual ability to predict unemployment risk, job separation rates, or within-occupation skill change (see Fig. 4) suggest that more research is required to converge on good solutions with practical use in policy making. The significant predictive performance of the ensemble model (see Fig. 3) demonstrates that existing scores identify key factors when taken together thus offering a pathway forward.

A better understanding of why AI exposure scores perform differently depending on the state, year, and occupation (see Fig. 5) could yield further progress. For example, the high performance of scores from Webb’s study (11) in California may be expected because of the study’s focus on information technology and the technology industry’s presence in Silicon Valley. Similarly, the scores used in this study were published across the past decade, and their association with labor outcomes across states and/or occupations varies over time. That is, we use scores from existing studies in the last decade to predict labor outcomes throughout the entire time period regardless of the study’s publication year (i.e. all scores are used for both prospectively and retrospectively). More research may identify composite AI exposure scores that leverage the methodological strengths of studies across multiple waves.

Our study has limitations. First, our analysis of unemployment risk used unemployment data for occupation groups (i.e. Standard Occupation Classification Major Occupations) when more granular occupation data would be preferred. Similarly, our analysis of job separation rates was limited to the aggregation of states rather than by occupations, industries, or firms within states. Despite these shortcomings, to the best of our knowledge, there are no better comprehensive, nationally representative data for describing unemployment risk or job separations. Refining data from the Department of Labor (20, 26) will spur dramatic improvements in predictions of technology and the future of work. Second, this study does not establish causality between AI exposure and labor outcomes because natural experiments are rare at the national scale. Similarly, none of the AI exposure studies mentioned in this article establish causality, instead offering a method for conceptualizing and predicting job disruptions from automation. Endogeneity exists because most technologies grow enough to impact the national labor system only if they fill some existing demand in the labor market. Although our study fails to control for every potential confound, our analyses do control for year (i.e. long-term trends), month (i.e. seasonality), and state (i.e. geographical) fixed effects where appropriate. Many factors, such as workers’ self-selection into different types of work or increased global competition (43, 44), will confound the relationship between AI exposure and unemployment risk through skills or education requirements and, thus, would be at least partially controlled for in our analysis.

In conclusion, we have demonstrated that many concerning labor outcomes can be predicted with an ensemble model of AI exposure scores. Examples include unemployment risk, skill change per occupation, and job separation rates by state. While policymakers strive to prepare workers for AI and the future of work, new methods are required to holistically quantify which workers have exposure to technology and the risk of detrimental labor outcomes.

## Supplementary Material

pgaf107_Supplementary_Data

## Data Availability

All data in this study are publicly available from the US Department of Labor or from existing peer-reviewed studies. All data and code used in this analysis will be available for download from https://github.com/mrfrank8176/unemploymentRisk upon publication (45).

## Notes

a Here, we limit to data between January 2010 and December 2019 to avoid months with out-of-equilibrium employment dynamics during the 2020 COVID Recession. Unemployment risk by occupation and state is calculated monthly, but employment statistics are only updated annually; so we compare the average annual unemployment risk for each occupation–state pair to the annual employment share for that occupation in that state.

b Here, BLS LAUS total unemployment is available monthly. We compare monthly total unemployment rates by state to unemployment risk by occupation–state pair. Unless otherwise noted, we limit to data between January 2010 and December 2019 to avoid months with out-of-equilibrium employment dynamics during the 2020 COVID Recession.

## Preprints

This manuscript was posted on a preprint: https://arxiv.org/abs/2308.02624.
