# Supplementary material for: AI exposure predicts unemployment risk: A new approach to technology-driven job loss
Source: PNAS Nexus. 2025 Apr 2;4(4):pgaf107. doi: 10.1093/pnasnexus/pgaf107 (PMC11983276; doi:10.1093/pnasnexus/pgaf107)
Supplement: pgaf107_Supplementary_Data [file pgaf107_supplementary_data.pdf]

Supplementary Materials: AI exposure predicts unemployment risk: A new approach to technology-driven job loss

Morgan R. Frank[1,2,3,4,*], Yong-Yeol Ahn[4,5], and Esteban Moro[3,6,7]

[1]*Department of Informatics and Networked Systems, University of Pittsburgh, Pittsburgh, PA 15216 USA*
[2]*Digital Economy Lab, Institute for Human-Centered Artificial Intelligence, Stanford University, Stanford, CA 94305 USA*
[3]*Media Laboratory, Massachusetts Institute of Technology, Cambridge, MA, 02139 USA*
[4]*Connection Science, Massachusetts Institute of Technology, Cambridge, MA, USA*
[5]*Center for Complex Networks and Systems Research, Luddy School of Informatics, Computing, and Engineering, Indiana University, Bloomington, IN, USA*
[6]*Department of Mathematics & GISC, Universidad Carlos III de Madrid, 28911 Leganes, Spain*
[7]*Network Science Institute, Northeastern University, Boston, MA, USA*
[*]*To whom correspondence should be addressed. Email: mrfrank@pitt.edu*

Contents

1 Estimating Technology Exposure **1**

2 Technology Exposure by State **2**

3 Quantifying Occupations' Unemployment Risk and States' Job Separations **2**

4 O*NET Principal Component Analysis **3**

5 Automation Model Shapley Values for Predicting Unemployment Risk **5**

6 Unemployment Risk by Occupation, State, & Month **8**

7 State Unemployment Rates **14**

8 State Job Separation Rates **15**

9 Within-Occupation Skill Change **18**

10 Predicting Wage Bill Change **20**

1 Estimating Technology Exposure

Estimations of technology exposure have evolved over the last decade. The first wave of theoretical studies adapted a production function (*1, 2*) to worker productivity in the presence of automating technologies, arguing that college-educated cognitive high-skill workers were complemented by technology, including computers, while manual low-skill workers were substituted by technologies like robotics. However, current AI technologies threaten cognitive workers as well. As examples, consider that AI surpasses human performance at predicting heart attacks (*3*) or

computer vision applications in radiology (*4–6*) and neighborhood safety (*7*). The modern, skill-biased technological change framework (*8*) further argues that both routine manual and cognitive work are ripe for automation, although cognitive workers will tend toward greater productivity with technology while manual workers will tend towards labor substitution (e.g., in manufacturing (*9*)).

The second wave of studies considered each occupation as a bundle of skill requirements and job tasks. Beyond describing occupations as cognitive or routine, occupations' granular skill requirements are considered, for example, by using the US Bureau of Labor Statistics (BLS) O*NET database (*10–12*). An Oxford University study (*13*) subjectively identified "fully automatable" and "not automatable" occupations combined with a subset of O*NET variables representing perception, manipulation, creativity, and social intelligence requirements of occupations. They used a logistic regression to assign a "probability of computerisation" to each remaining US occupation. Alarmingly, they claimed that 47% of US employment had high risk of computerization. However, the study only compared their estimates with occupations' education requirements and wages, thus leaving whether this exposure creates unemployment or alters skill demands unclear. A competing study directly estimated the automation risk of skills from the Programme for the International Assessment of Adult Competencies (PIAAC) survey enabling a direct assessment of the technological exposure of occupations in OECD countries (*14*), finding that only 9% of US workers had high automation risk. Subsequent studies used these occupation estimates in a variety of contexts; for example, finding that automation will affect 35% of employment in Finland (*15*), 59% of employment in Germany (*16*), 45 to 60% of employment across Europe (*17*), and that small US cities face greater impact from automation (*18*). To meet the demand for occupation-level automation estimates, the BLS added a Degree of Automation score to occupation profiles in the 2016 O*NET database.

The most-recent third wave of studies directly connects specific technological capabilities to occupations' job tasks to assess each occupation's exposure (i.e., a task-based approach (*19*)). One study surveyed machine learning (ML) experts on the characteristics of tasks that are suitable for ML and produced a Suitability for ML score for US occupations (*20*). Another study surveyed gig workers to establish connections between AI application capabilities and occupations (*21*). A more recent study used natural language processing to connect technology patents to job tasks (*22*). Although motivated by the risk of technological unemployment, these studies argue that AI exposure will mostly result in labor reorganization through wealth inequality or changing skill demands without major changes to unemployment.

2 Technology Exposure by State

We study monthly job separation and total unemployment rates by state using the Job Openings and Labor Turnover Survey (JOLTS) and Local Area Unemployment Statistics (LAUS) from the US Bureau of Labor Statistics (BLS). Unfortunately, these data are not stratified by state and occupation, but they are representative of each state's monthly economy. Given a per-occupation technology exposure score $exposure(j)$, we calculate the aggregate exposure for state s in year y according to

$$exposure(s, y) = \sum_{j \in SOC} exposure(j) \cdot share_{s,y}(j) \tag{1}$$

where j is a six-digit Standard Occupation Classification code and $share_{s,y}(j)$ is the share of employment associated with occupation j in that state and year according to the BLS.

3 Quantifying Occupations' Unemployment Risk and States' Job Separations

This study uses federal government data spanning 2010 through 2020 (except where a different time frame is noted). The US Bureau of Labor Statistics (BLS) Local Area Unemployment Statistics (LAUS) provides total

unemployment rates, and the BLS Job Openings and Labor Turnover Survey (JOLTS) provides total job separation rates (i.e., fires and quits) by state and month. These data capture the aggregate labor dynamics in each state but obfuscate the heterogeneous dynamics of workers across occupations. Evaluating technology exposure additionally requires high-resolution per-occupation data about unemployment risk and changes to skill demands. We meet this requirement with data describing occupations according to the Standard Occupation Classification (SOC) system. The SOC is used by the US Department of Labor as a standardized taxonomy of occupations. Each of about 700 occupations are specified by a unique six-digit code where the first two digits also indicate the occupation's *major* occupation group (hereafter, "major occupation"). For example, the BLS Occupational Employment and Wage Statistics (OEWS) details the employment distribution over six-digit occupations in each state each year. The BLS also provides the O*NET database, which provides annual profiles of the skills, knowledge, abilities, and work contexts required by each six-digit SOC code.

These data include occupation employment and wages but lack insights into unemployment by occupation. We overcome this barrier using data from the US Department of Education and Training Administration (DETA). The DETA reports characteristic unemployment data from each US state's unemployment insurance department detailing the most recent major occupation of every benefit recipient in every state every month. Using this data, we calculate unemployment risk ($p(unemp|soc, s, t)$) for each occupation in each state in each month (see main text for description). We provide distributions of unemployment risk estimates in Figure S1. We compare each US state's total unemployment from BLS LAUS to the total number of unemployment recipients from the DETA data (i.e., we measure the Pearson correlation between log-transformed values. See Table S1). In general, both quantities are highly correlated.

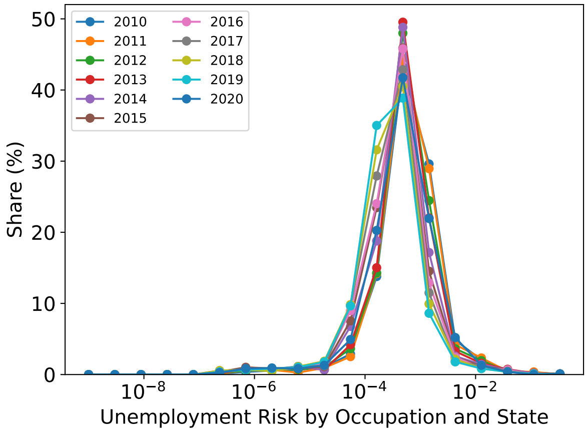

Figure S1: Annual distributions of occupation unemployment risk. The median of monthly risk scores by Major SOC code and state within each year (i.e., median taken over monthly values within each year).

4 O*NET Principal Component Analysis

We quantify occupation's skill requirements using the US Bureau of Labor Statistic's (BLS) O*NET skills database. These occupation skill profiles result from nationally-representative worker surveys covering over 700 different job titles from the Standard Occupation Classification (SOC) system and details the importance of 120 to 230 examples of workplace knowledge, abilities, work activities, and skills (henceforth, "skills") in each year from 2010 through 2020. Each occupation is identified by a unique six-digit SOC code where the first two-digits describe the occupation's Major Occupation type. We use $onet_{y,j,s} \in [0, 1]$ to denote the importance of skill $s \in S$ to occupation $j \in J$ in year y such that $onet_{y,j,s} = 1$ identifies an essential skill and $onet_{y,j,s} = 0$ indicates an

State	Pearson Corr.
NC	0.953
NH	0.928
AR	0.927
ID	0.919
IN	0.911
PA	0.911
FL	0.908
HI	0.900
DE	0.896
NE	0.896
TN	0.886
WI	0.885
AZ	0.883
KS	0.882
AK	0.880
MO	0.879
SD	0.875
OR	0.873
MD	0.873
WY	0.870
ND	0.860
OK	0.859
AL	0.855
WA	0.852
NY	0.848
NM	0.848
WV	0.842
MA	0.840
MS	0.831
UT	0.821
LA	0.816
SC	0.815
OH	0.812
CO	0.808
VT	0.807
VA	0.806
MT	0.806
IA	0.801
IL	0.797
NJ	0.796
NV	0.794
MN	0.793
MI	0.773
ME	0.773
RI	0.766
GA	0.744
CT	0.738
KY	0.724
CA	0.721
TX	0.705

Table S1: Within each state, monthly total unemployment from BLS LAUS is tightly correlated with the total number of unemployment recipients in our data used to calculate unemployment risk. All correlations have p-values below 0.001.

irrelevant skill. We simplify these annual occupation skill profiles using principal component analysis (PCA). Figure S2 demonstrates the variation in skill requirements captured by PCA as a function of the number of principal components included in the analysis. Throughout this study, we use the first ten principal components to control for occupations' skill requirements. The first ten principal components account for 96% of the total variation in occupation's skill requirements across all years of the O*NET data. These principal components are used in our analysis of unemployment risk to control for occupations' skill requirements.

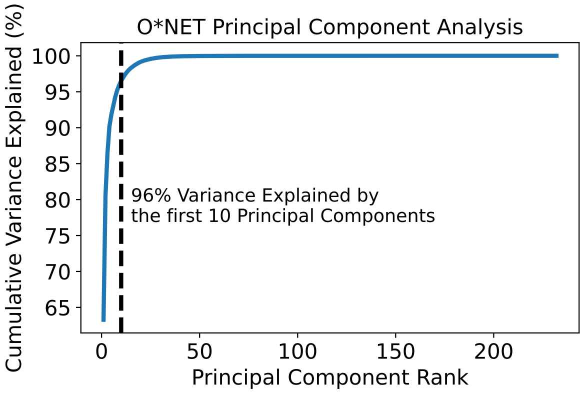

Figure S2: O*NET variance explained by the number of principal components included. In this study, we use the first ten principal components which cumulatively account for 96% of the overall variation in occupations' skill requirements by year according to O*NET.

5 Automation Model Shapley Values for Predicting Unemployment Risk

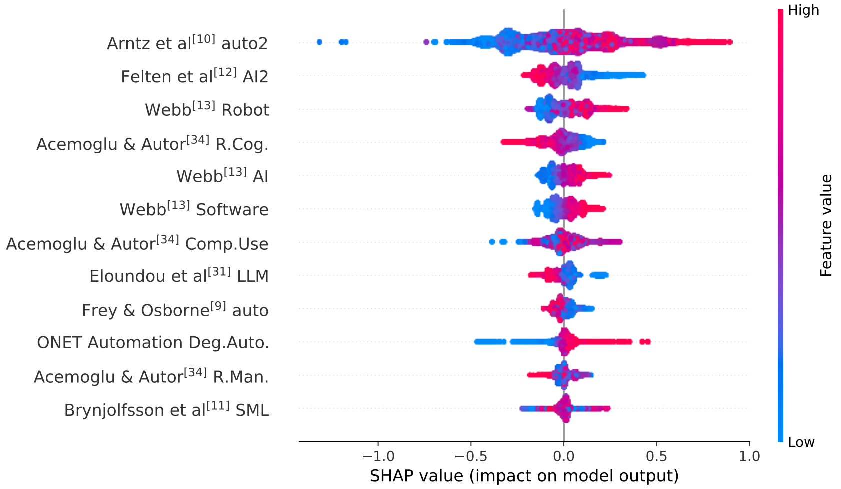

Figure S3: A Beeswarm plot demonstrating the Shapley value (x-axis) for each automation score (y-axis) when predicting unemployment risk by occupation, state, and month. The color indicates how the automation score relates to it's Shapley value across data points.

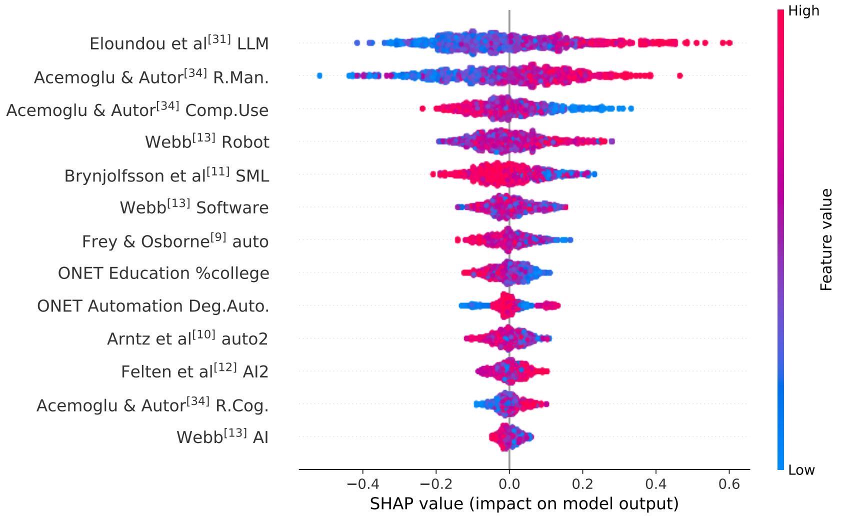

Figure S4: A Beeswarm plot demonstrating the Shapley value (x-axis) for each automation score (y-axis) when predicting total separations by state and month. The color indicates how the automation score relates to it's Shapley value across data points.

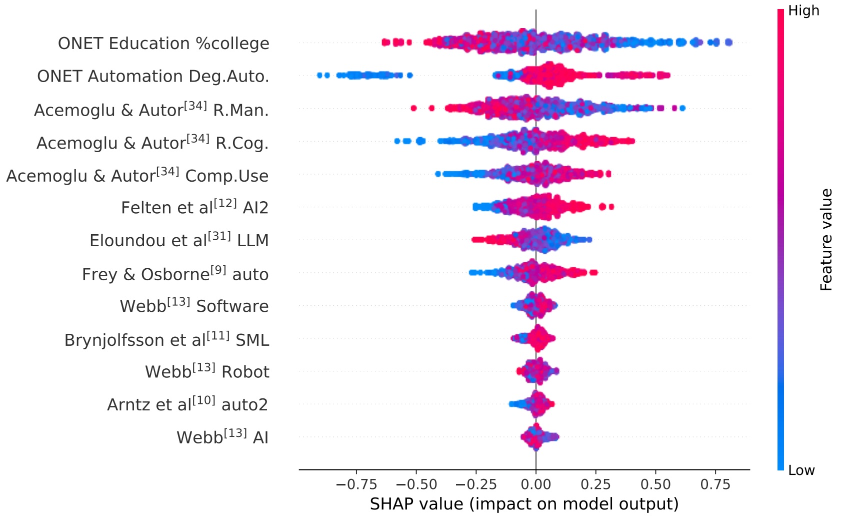

Figure S5: A Beeswarm plot demonstrating the Shapley value (x-axis) for each automation score (y-axis) when predicting total unemployment rate by state and month. The color indicates how the automation score relates to it's Shapley value across data points.

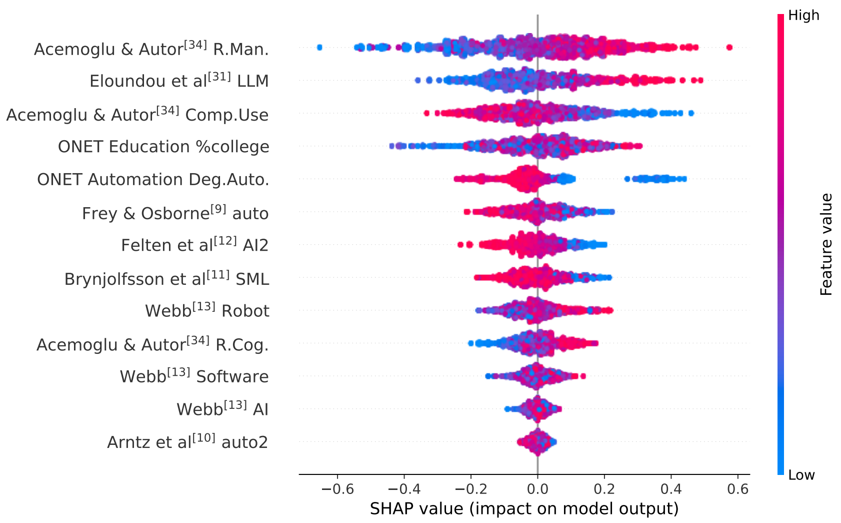

Figure S6: A Beeswarm plot demonstrating the Shapley value (x-axis) for each automation score (y-axis) when predicting total job quits by state and month. The color indicates how the automation score relates to it's Shapley value across data points.

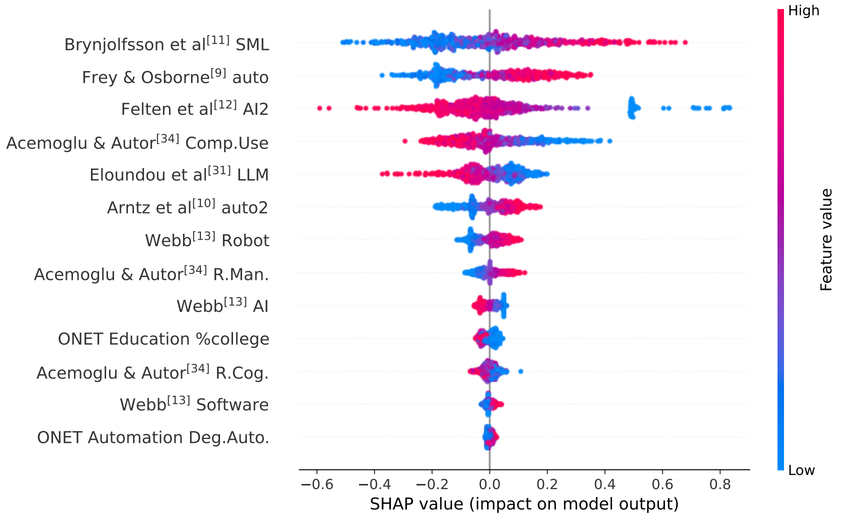

Figure S7: A Beeswarm plot demonstrating the Shapley value (x-axis) for each automation score (y-axis) when predicting within-occupation skill change by occupation. The color indicates how the automation score relates to it's Shapley value across data points.

6 Unemployment Risk by Occupation, State, & Month

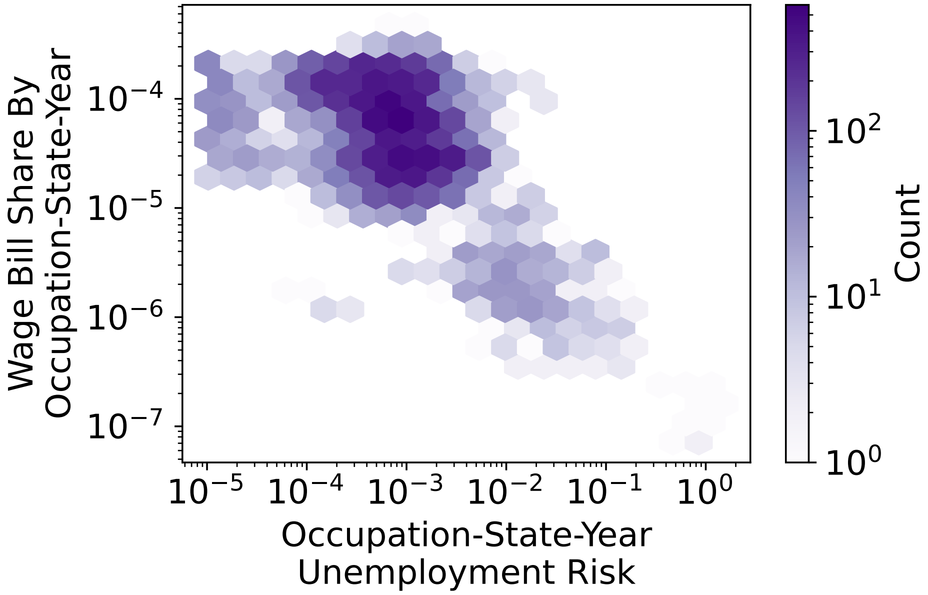

Figure S8: Using occupation wage data, we observe a negative correlation between unemployment risk and wages. BLS OEWS describes occupation wages for each six-digit SOC code, state, and year. However, unemployment risk is only calculated for each two-digit SOC code (i.e., Major Occupation), state, and year. We sum six-digit SOC wage bill values by state to calculate wage bill values for two-digit SOC codes in each state in each year.

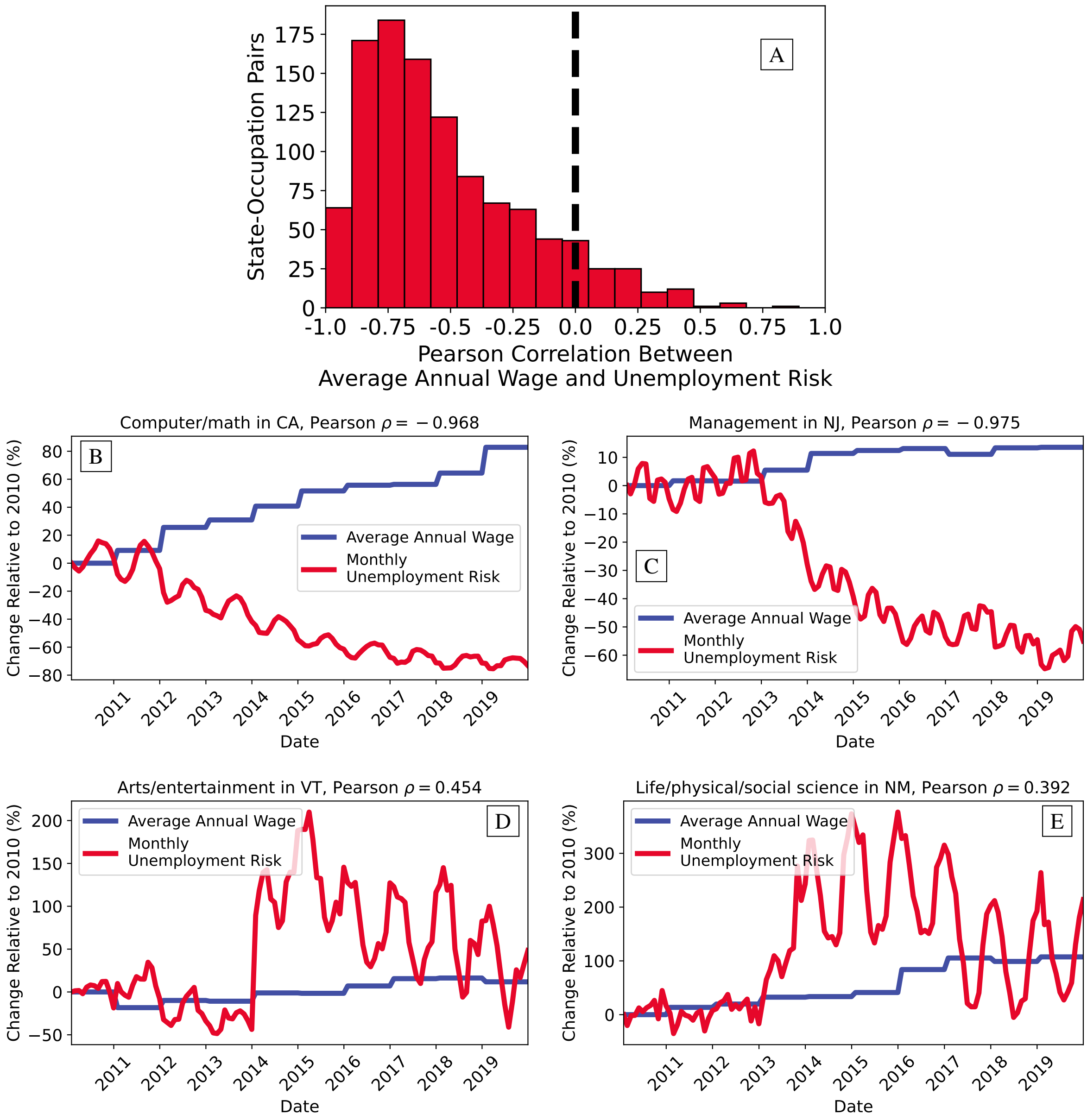

Figure S9: **(A)** By occupation and state, temporal shifts in wages are not predictive of unemployment risk in general. For most state-occupation pairs, increasing wages correspond to decreasing unemployment, but not always. **(B) & (C)** Examples of occupation-state pairs where increasing annual wages corresponded to decreasing unemployment risk. **(D) & (E)** Examples of occupation-state pairs where increasing annual wages corresponded to increasing unemployment risk.

Dependent Variable: Log_{10} Unemployment Risk by Occupation, Month, & State									
Variable	Model 1	Model 2	Model 3	Model 4	Model 5	Model 6	Model 7	Model 8	Model 9
Acemoglu & Autor (*8*) Comp.Use	0.048^{***}								
Acemoglu & Autor (*8*) R.Cog.	-0.121^{***}								
Acemoglu & Autor (*8*) R.Man.	0.185^{***}								
ONET Education %college		-0.135^{***}							
Frey & Osborne (*13*) auto			0.015^{***}						
Arntz et al (*14*) auto2				0.318^{***}					
ONET Automation Deg.Auto.					0.145^{***}				
Brynjolfsson et al (*20*) SML						0.074^{***}			
Felten et al (*21*) AI2							-0.118^{***}		
Webb (*22*) AI								0.330^{***}	
Webb (*22*) Robot								0.409^{***}	
Webb (*22*) Software								-0.291^{***}	
Eloundou et al (*23*) LLM									-0.163^{***}
R^2	0.027	0.018	0.000	0.101	0.021	0.006	0.015	0.087	0.027
adj. R^2	0.027	0.018	0.000	0.101	0.021	0.006	0.015	0.087	0.027
$p_{val} < 0.1^{*}, p_{val} < 0.01^{**}, p_{val} < 0.001^{***}$									

Table S2: Linear regression analysis of occupations' technology exposure and unemployment risk. Data varies by Major SOC code, state, and month from January 2010 through 2020 for a total of 140,274 data points. All variables were centered and standardized before analysis, thus eliminating units from the various exposure variables.

Dependent Variable: Log_{10} Unemployment Risk by Occupation, Month, & State										
Variable	Model 1	Model 2	Model 3	Model 4	Model 5	Model 6	Model 7	Model 8	Model 9	Model 10
Acemoglu & Autor (*8*) Comp.Use		0.728^{***}								0.493^{***}
Acemoglu & Autor (*8*) R.Cog.		-0.511^{***}								-0.192^{***}
Acemoglu & Autor (*8*) R.Man.		0.000								0.000
Frey & Osborne (*13*) auto			-0.608^{***}							-0.314^{***}
Arntz et al (*14*) auto2				0.231^{***}						0.364^{***}
ONET Automation Deg.Auto.					0.064^{***}					0.000
Brynjolfsson et al (*20*) SML						-0.099^{***}				-0.152^{***}
Felten et al (*21*) AI2							-0.156^{***}			0.000
Webb (*22*) AI								0.507^{***}		0.055^{***}
Webb (*22*) Robot								0.410^{***}		0.518^{***}
Webb (*22*) Software								-0.038^{***}		-0.000
Eloundou et al (*23*) LLM									-0.139^{***}	-0.044^{***}
ONET Education %college	0.039^{***}	-0.115^{***}	-0.032^{***}	0.060^{***}	0.000	0.097^{***}	0.036^{***}	-0.132^{***}	0.081^{***}	-0.018^{***}
O*NET PCA	Yes	Yes	Yes	Yes	Yes	Yes	Yes	Yes	Yes	Yes
Year F.E.	Yes	Yes	Yes	Yes	Yes	Yes	Yes	Yes	Yes	Yes
Month F.E.	Yes	Yes	Yes	Yes	Yes	Yes	Yes	Yes	Yes	Yes
State F.E.	Yes	Yes	Yes	Yes	Yes	Yes	Yes	Yes	Yes	Yes
R^2	0.574	0.688	0.693	0.581	0.576	0.581	0.586	0.661	0.583	0.755
adj. R^2	0.574	0.688	0.693	0.581	0.576	0.581	0.586	0.661	0.583	0.755
$p_{val} < 0.1^{*}, p_{val} < 0.01^{**}, p_{val} < 0.001^{***}$										

Table S3: LASSO regression analysis of occupations' technology exposure and unemployment risk. Data varies by Major SOC code, state, and month from January 2010 through 2020 for a total of 140,274 data points. All variables were centered and standardized before analysis, thus eliminating units from the various AI exposure variables.

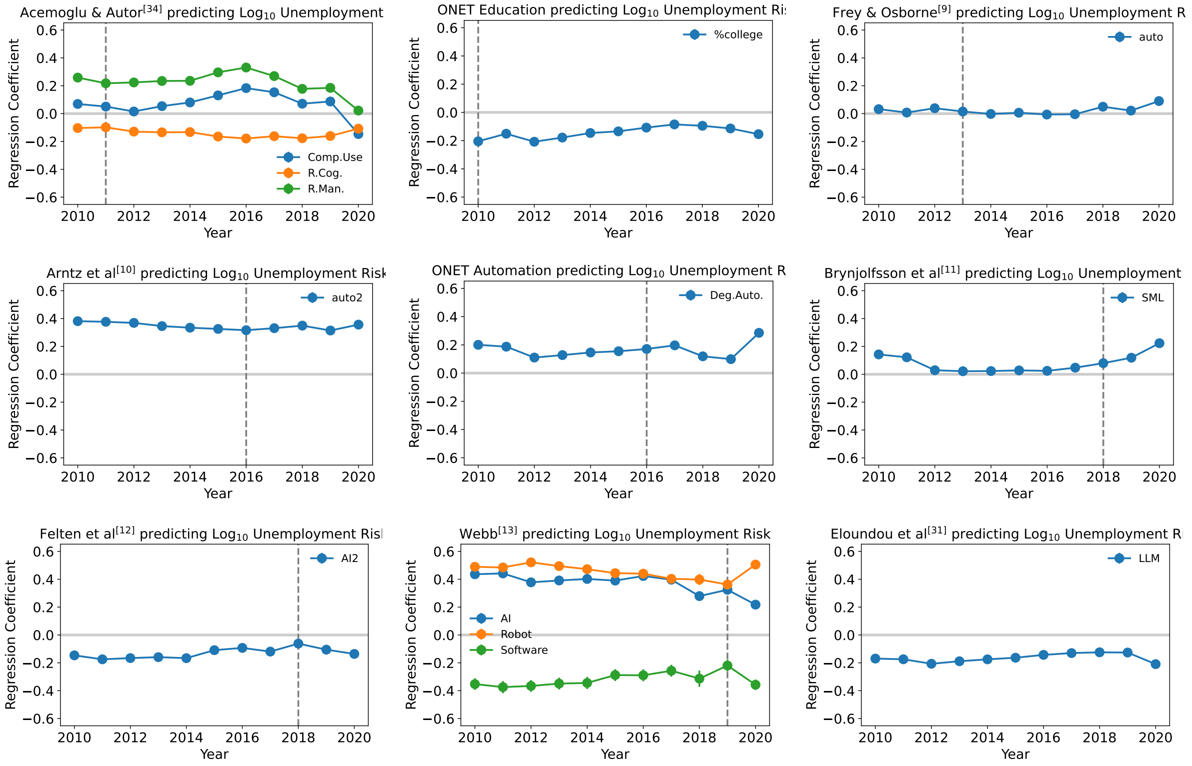

Figure S10: Variables' regression coefficient estimate with $\log_{10}$ unemployment risk as the dependent variable. Bars represent 95% confidence intervals. Independent regressions with month and state fixed effects were performed for each year. All variables were centered and standardized before analysis, thus eliminating units from the various AI exposure variables.

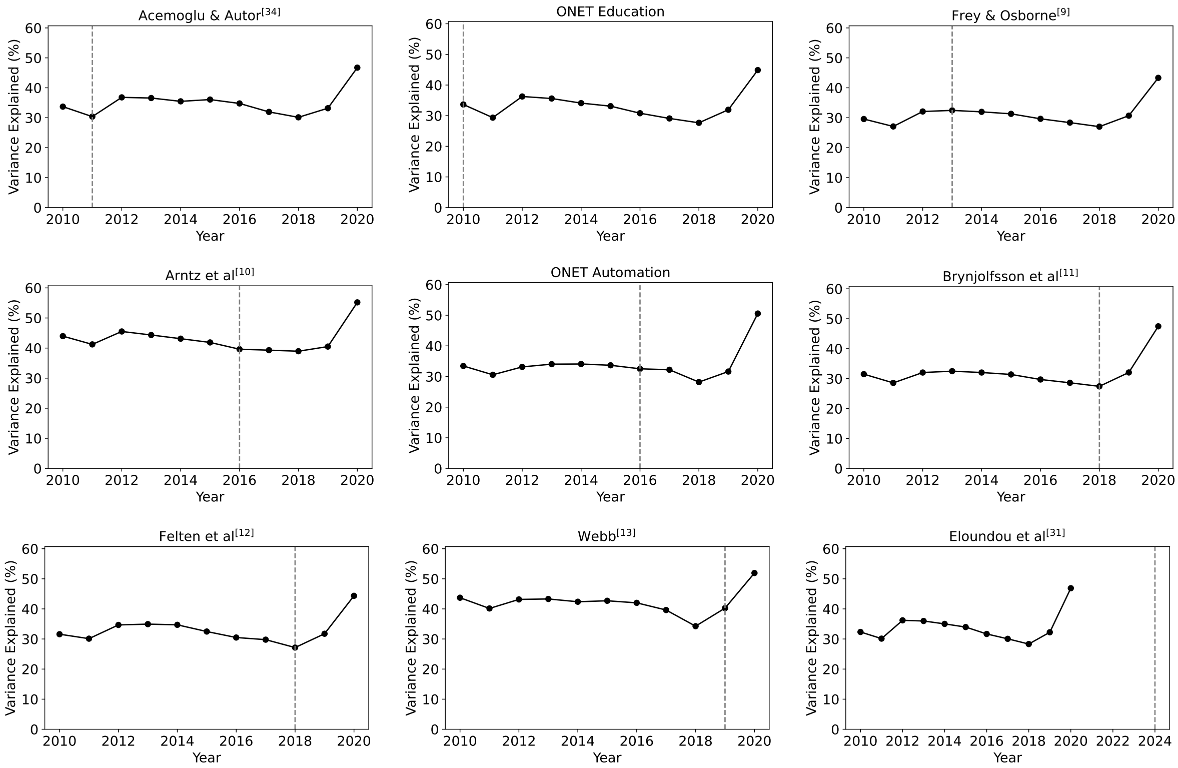

Figure S11: $\log_{10}$ unemployment risk variance explained by each model. Independent regressions with month and state fixed effects were performed for each year. All variables were centered and standardized before analysis, thus eliminating units from the various AI exposure variables. Vertical dashed line represents the year that AI exposure variables were made available.

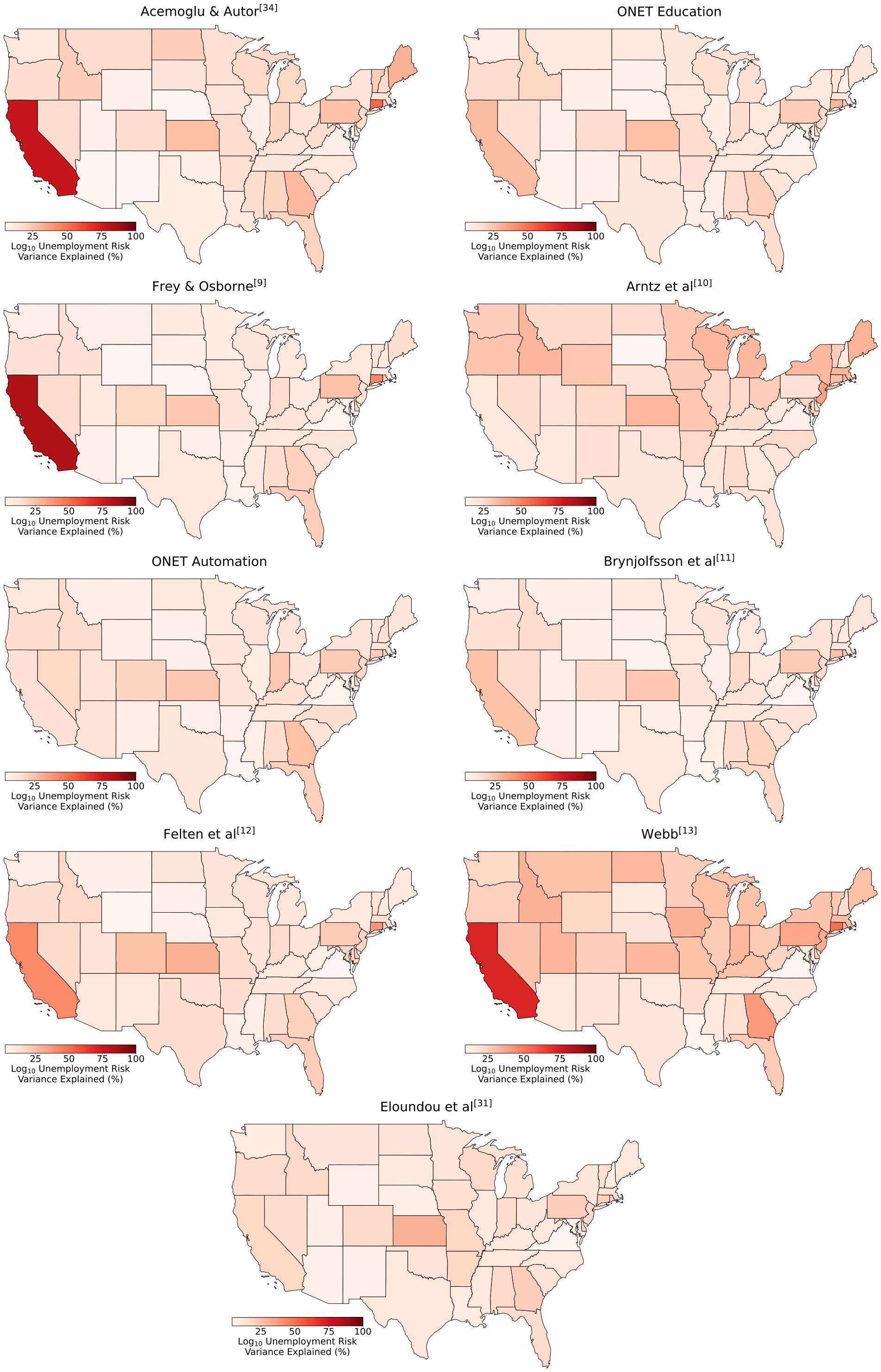

Figure S12: $\log_{10}$ unemployment risk variance explained by each model. Independent regressions with month and year fixed effects were performed for each state.

7 State Unemployment Rates

Dependent Variable: Log_{10} Unemployment Rate by State & Month									
Variable	Model 1	Model 2	Model 3	Model 4	Model 5	Model 6	Model 7	Model 8	Model 9
Acemoglu & Autor (*8*) Comp.Use	0.015								
Acemoglu & Autor (*8*) R.Cog.	0.460^{***}								
Acemoglu & Autor (*8*) R.Man.	-0.347^{***}								
ONET Education %college		-0.088^{***}							
Frey & Osborne (*13*) auto			0.239^{***}						
Arntz et al (*14*) auto2				0.151^{***}					
ONET Automation Deg.Auto.					0.323^{***}				
Brynjolfsson et al (*20*) SML						0.166^{***}			
Felten et al (*21*) AI2							0.252^{***}		
Webb (*22*) AI								-0.023	
Webb (*22*) Robot								0.048	
Webb (*22*) Software								0.082^{*}	
Eloundou et al (*23*) LLM									-0.074^{***}
R^2	0.175	0.008	0.057	0.023	0.104	0.028	0.063	0.012	0.005
adj. R^2	0.174	0.008	0.057	0.023	0.104	0.027	0.063	0.012	0.005
$p_{val} < 0.1^{*}, p_{val} < 0.01^{**}, p_{val} < 0.001^{***}$									

Table S4: Linear regression analysis of states' technology exposure and total unemployment rates according to LAUS. Data varies by state, and month from January 2010 through 2020 for a total of 6,600 data points. All variables were centered and standardized before analysis thus eliminating units from the various exposure variables.

Dependent Variable: Log_{10} Unemployment Rate by State & Month											
Variable	Model 1	Model 2	Model 3	Model 4	Model 5	Model 6	Model 7	Model 8	Model 9	Model 10	Model 11
Acemoglu & Autor (*8*) Comp.Use		-0.108^{***}									0.047
Acemoglu & Autor (*8*) R.Cog.		0.175^{***}									0.373^{***}
Acemoglu & Autor (*8*) R.Man.		-0.196^{***}									-0.619^{***}
ONET Education Frey & Osborne (*13*) auto				-0.019							-0.091^{*}
Arntz et al (*14*) auto2					-0.018						-0.156^{***}
ONET Automation Deg.Auto.						-0.394^{***}					-0.063
Brynjolfsson et al (*20*) SML							-0.239^{***}				-0.645^{***}
Felten et al (*21*) AI2								-0.193^{***}			-0.127^{***}
Webb (*22*) AI									0.102^{**}		1.223^{***}
Webb (*22*) Robot									0.195^{***}		0.857^{***}
Webb (*22*) Software									-0.188^{***}		-0.758^{***}
Eloundou et al (*23*) LLM										-0.120^{***}	-0.070^{*}
$\log_{10}$ Wage Bill ($)	0.241^{***}	0.221^{***}	0.279^{***}	0.237^{***}	0.236^{***}	0.236^{***}	0.231^{***}	0.213^{***}	0.270^{***}	0.269^{***}	0.135^{***}
Year F.E.	Yes	Yes	Yes	Yes	Yes	Yes	Yes	Yes	Yes	Yes	Yes
Month F.E.	Yes	Yes	Yes	Yes	Yes	Yes	Yes	Yes	Yes	Yes	Yes
R^2	0.598	0.618	0.605	0.598	0.598	0.603	0.601	0.606	0.600	0.604	0.699
adj. R^2	0.597	0.616	0.604	0.597	0.597	0.602	0.600	0.605	0.599	0.602	0.698
$p_{val} < 0.1^{*}, p_{val} < 0.01^{**}, p_{val} < 0.001^{***}$											

Table S5: Multiple linear regression analysis of states' technology exposure and total unemployment rates each month according to LAUS. Data varies by state and month from January 2010 through 2020 for a total of 6,600 data points. All variables were centered and standardized before analysis thus eliminating units from the various AI exposure variables. We are unable to control for state fixed effects in this analysis, so we instead control for the size of each states' economy according to their total wage bill (i.e., total wages paid).

8 State Job Separation Rates

Dependent Variable: Log_{10} Total Separations by State & Month									
Variable	Model 1	Model 2	Model 3	Model 4	Model 5	Model 6	Model 7	Model 8	Model 9
Acemoglu & Autor (*8*) Comp.Use	0.073***								
Acemoglu & Autor (*8*) R.Cog.	-0.128***								
Acemoglu & Autor (*8*) R.Man.	0.394***								
ONET Education %college		-0.196***							
Frey & Osborne (*13*) auto			-0.038**						
Arntz et al (*14*) auto2				-0.019					
ONET Automation Deg.Auto.					-0.112***				
Brynjolfsson et al (*20*) SML						-0.111***			
Felten et al (*21*) AI2							-0.074***		
Webb (*22*) AI								-0.202***	
Webb (*22*) Robot								0.018	
Webb (*22*) Software								0.148***	
Eloundou et al (*23*) LLM									0.096***
R^2	0.092	0.038	0.001	0.000	0.013	0.012	0.006	0.018	0.009
adj. R^2	0.092	0.038	0.001	0.000	0.012	0.012	0.005	0.017	0.009
$p_{val} < 0.1^{*}, p_{val} < 0.01^{**}, p_{val} < 0.001^{***}$									

Table S6: Linear regression analysis of states' technology exposure and total job separation rate according to JOLTS. Data varies by state, and month from January 2010 through 2020 for a total of 6,600 data points. All variables were centered and standardized before analysis thus eliminating units from the various exposure variables.

Dependent Variable: Log_{10} Total Separations by State & Month											
Variable	Model 1	Model 2	Model 3	Model 4	Model 5	Model 6	Model 7	Model 8	Model 9	Model 10	Model 11
Acemoglu & Autor (*8*) Comp.Use		0.004									0.290***
Acemoglu & Autor (*8*) R.Cog.		0.066***									0.121***
Acemoglu & Autor (*8*) R.Man.		0.117***									-0.062*
ONET Education %college			-0.213***								-0.212***
Frey & Osborne (*13*) auto				0.244***							0.588***
Arntz et al (*14*) auto2					0.197***						-0.259***
ONET Automation Deg.Auto.						0.042					-0.091
Brynjolfsson et al (*20*) SML							0.148***				-1.154***
Felten et al (*21*) AI2								0.281***			0.397***
Webb (*22*) AI									-0.021		0.247***
Webb (*22*) Robot									0.245***		0.696***
Webb (*22*) Software									0.011		-0.325***
Eloundou et al (*23*) LLM										-0.121***	0.136**
$\log_{10}$ Wage Bill ($)	-0.259***	-0.220***	-0.201***	-0.199***	-0.205***	-0.258***	-0.253***	-0.218***	-0.191***	-0.231***	-0.169***
Year F.E.	Yes	Yes	Yes	Yes	Yes	Yes	Yes	Yes	Yes	Yes	Yes
Month F.E.	Yes	Yes	Yes	Yes	Yes	Yes	Yes	Yes	Yes	Yes	Yes
R^2	0.379	0.401	0.395	0.401	0.393	0.379	0.380	0.395	0.404	0.384	0.457
adj. R^2	0.376	0.398	0.393	0.399	0.391	0.376	0.377	0.393	0.401	0.382	0.454
$p_{val} < 0.1^{*}, p_{val} < 0.01^{**}, p_{val} < 0.001^{***}$											

Table S7: Multiple linear regression analysis of states' technology exposure and total job separation rates each month according to JOLTS. Data varies by state and month from January 2010 through 2020 for a total of 6,600 data points. All variables were centered and standardized before analysis thus eliminating units from the various AI exposure variables. We are unable to control for state fixed effects in this analysis, so we instead control for the size of each states' economy according to their total wage bill (i.e., total wages paid).

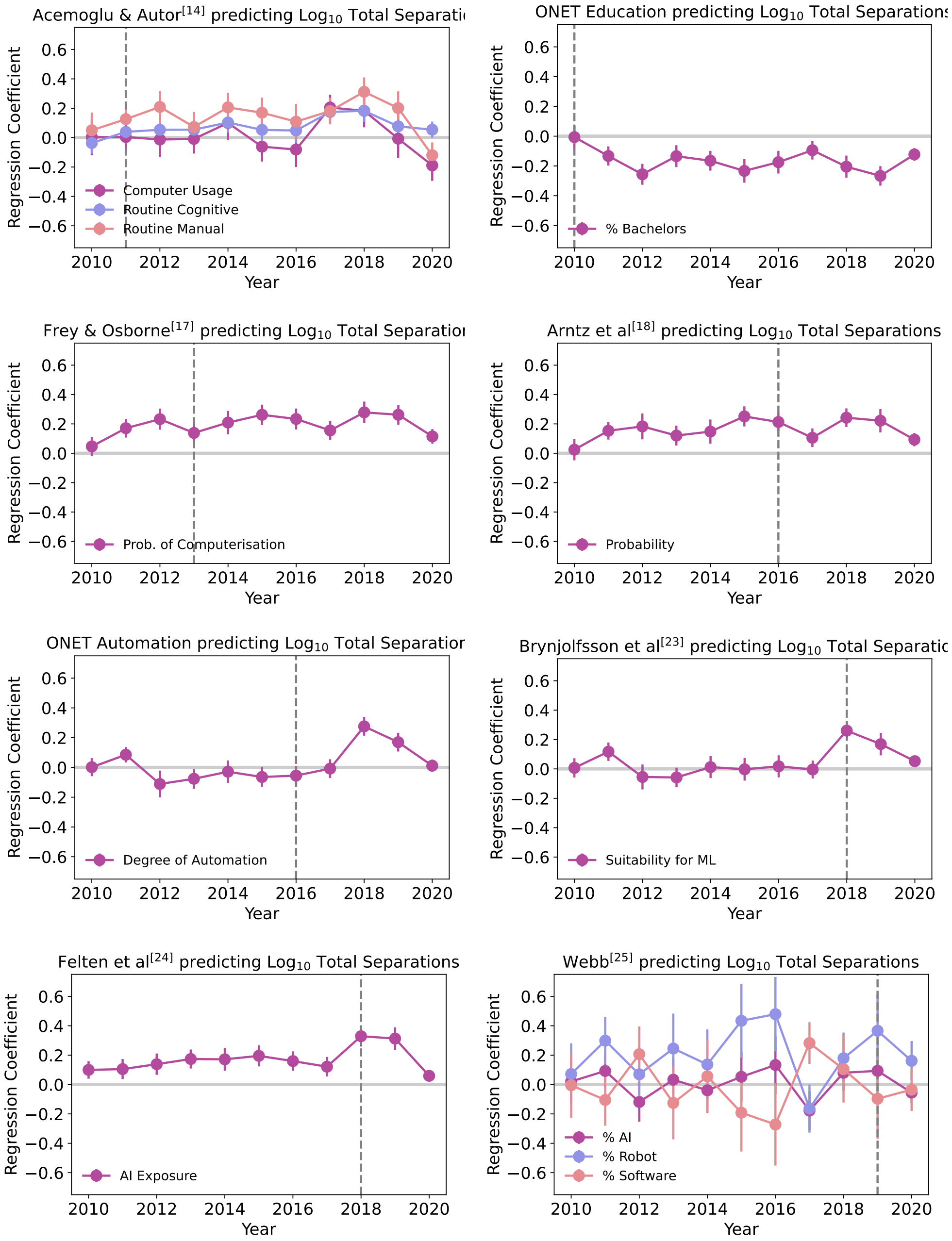

Figure S13: Variables' regression coefficient estimate with monthly $\log_{10}$ total job separations as the dependent variable. Bars represent 95% confidence intervals. Independent regressions with month fixed effects and were performed for each year while controlling for states' total wage bill (i.e., total wages paid). All variables were centered and standardized before analysis thus eliminating units from the various AI exposure variables.

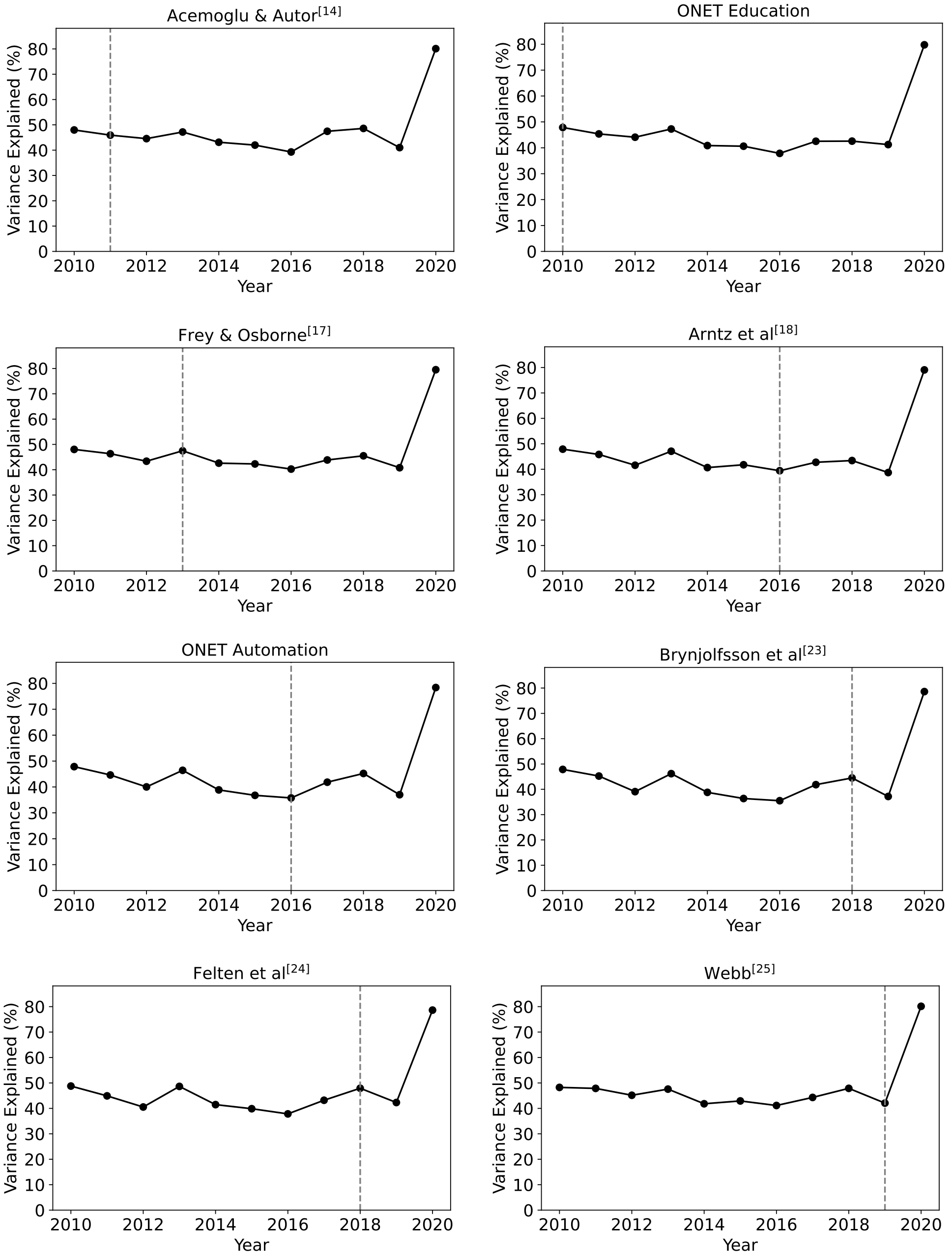

Figure S14: Monthly $\log_{10}$ total job separations variance explained by each model. Independent regressions with month fixed effects and were performed for each year while controlling for states' total wage bill (i.e., total wages paid). All variables were centered and standardized before analysis thus eliminating units from the various AI exposure variables.

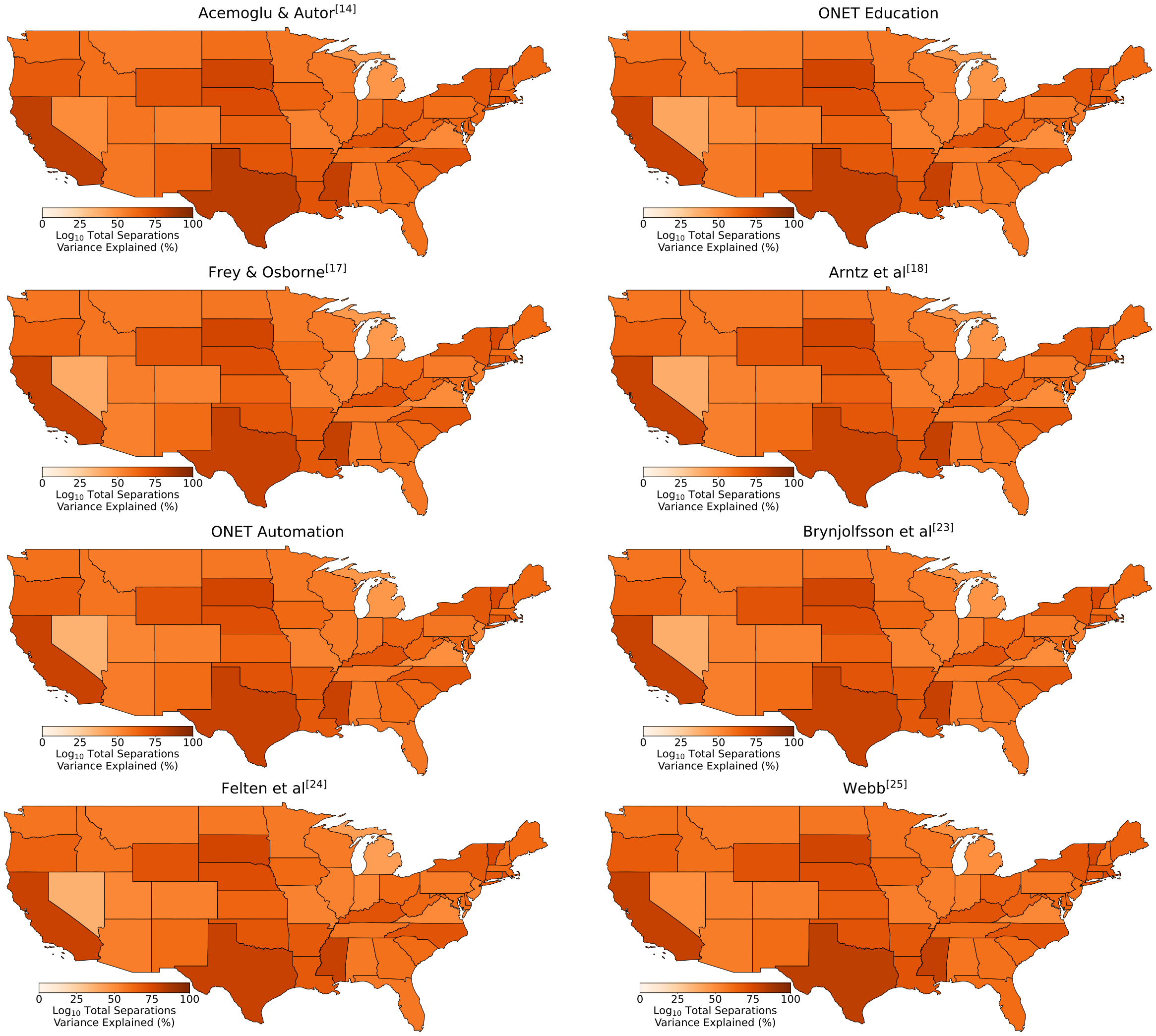

Figure S15: Monthly $\log_{10}$ total separations variance explained by each model. Independent regressions with month fixed effects were performed for each state while controlling for states' total wage bill (i.e., total wages paid).

9 Within-Occupation Skill Change

We explore within-occupation changes to skill demand using annual occupation skill profiles from the BLS O*NET database. After normalizing the varying Likert scales used across O*NET surveys, we obtain a skill vector for each occupation in each from 2010 through 2017. Let $onet_{j_y,s} \in [0,1]$ represent the real-valued importance of skill s to occupation j in year y such that $onet_{j_y,s} = 1$ identifies an essential skill and $onet_{j_y,s} = 0$ identifies an irrelevant skill. Inspired by earlier work (*24*), this data allows us to model each occupation as a vector of O*NET variables. O*NET is updated annually, but each occupation is only updated every five years through a rolling survey. That is, occupations are not updated each year. Therefore, for each six-digit Standard Occupation Classification (SOC) code j, we capture the within-occupation skill change in the year that j was updated in O*NET and compare the

updated skill profile to j's skill requirements in 2010 according to

$$\Delta skill(j_y, j_{2010}) = 1 - \frac{\sum_{s \in S} \min(onet_{j_y,s}, onet_{j_{2010},s})}{\sum_{s \in S} \max(onet_{j_y,s}, onet_{j_{2010},s})}. \tag{2}$$

Notice that eq (2) is one minus the Jaccard similarity and $\Delta skill \in [0, 1]$. We consider O*NET data only from 2010 through 2017 because the SOC taxonomy was updated in 2018. Figure S16 displays the distribution of skill change scores for the occupations that were updated in each year. Although we provide distributions for 2018, 2019, and 2020 in Fig. S16, all other analyses of skill change in this study are restricted to O*NET data from 2010 through 2017 because the SOC taxonomy was updated from 2018 on-wards (i.e., the list of occupation codes changed) which introduces the potential for skill changes to result from the merging or separation of 2010 SOC codes.

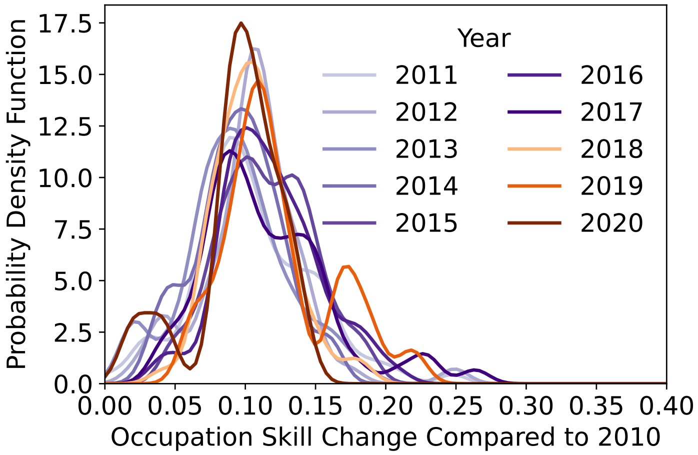

Figure S16: The annual distribution of within-occupation skill change compared to 2010. We consider only the occupations with skill profiles that were updated in each year. That is, each occupation contributes to the regression in only one year. We use 2011 through 2017 for the main analysis because the SOC taxonomy used to distinguish occupations was updated in 2018 thus introducing a potential confound to any longitudinal analysis.

Dependent Variable: Within-Occupation Skill Change compared to 2010 by Detailed Occupation									
Variable	Model 1	Model 2	Model 3	Model 4	Model 5	Model 6	Model 7	Model 8	Model 9
Acemoglu & Autor (*8*) Comp.Use	-0.275***								
Acemoglu & Autor (*8*) R.Cog.	0.062								
Acemoglu & Autor (*8*) R.Man.	0.069								
ONET Education %college		-0.216***							
Frey & Osborne (*13*) auto			0.345***						
Arntz et al (*14*) auto2				0.245***					
ONET Automation Deg.Auto.					0.050				
Brynjolfsson et al (*20*) SML						0.363***			
Felten et al (*21*) AI2							-0.256***		
Webb (*22*) AI								-0.203***	
Webb (*22*) Robot								0.261***	
Webb (*22*) Software								0.016	
Eloundou et al (*23*) LLM									-0.255***
R^2	0.108	0.047	0.119	0.060	0.002	0.132	0.065	0.081	0.065
adj. R^2	0.103	0.045	0.117	0.058	0.001	0.130	0.064	0.076	0.063
$p_{val} < 0.1^{*}, p_{val} < 0.01^{**}, p_{val} < 0.001^{***}$									

Table S8: Multiple linear regression analysis of within-occupation skill change compared to 2010 O*NET skill profiles. Data varies by six-digit SOC code from 2011 through 2017 for a total of 479 data points. All variables were centered and standardized before analysis thus eliminating potentially different units from the technology exposure scores.

Dependent Variable: Within-Occupation Skill Change compared to 2010 by Detailed Occupation											
Variable	Model 1	Model 2	Model 3	Model 4	Model 5	Model 6	Model 7	Model 8	Model 9	Model 10	Model 11
Acemoglu & Autor (*8*) Comp.Use		-0.214***									-0.048
Acemoglu & Autor (*8*) R.Cog.		-0.035									-0.066
Acemoglu & Autor (*8*) R.Man.		0.096									-0.002
ONET Education %college			-0.102*								0.052
Frey & Osborne (*13*) auto				0.220***							0.075
Arntz et al (*14*) auto2					0.075						0.028
ONET Automation Deg.Auto.						-0.057					-0.070
Brynjolfsson et al (*20*) SML							0.390***				0.349***
Felten et al (*21*) AI2								-0.344***			-0.277***
Webb (*22*) AI									-0.107		0.020
Webb (*22*) Robot									0.242**		0.064
Webb (*22*) Software									-0.022		-0.027
Eloundou et al (*23*) LLM										-0.266***	-0.190*
Year F.E.	Yes	Yes	Yes	Yes	Yes	Yes	Yes	Yes	Yes	Yes	Yes
Major SOC F.E.	Yes	Yes	Yes	Yes	Yes	Yes	Yes	Yes	Yes	Yes	Yes
R^2	0.251	0.274	0.257	0.272	0.255	0.254	0.292	0.306	0.268	0.272	0.363
adj. R^2	0.207	0.228	0.212	0.228	0.209	0.208	0.249	0.264	0.221	0.228	0.309
$p_{val} < 0.1^{*}, p_{val} < 0.01^{**}, p_{val} < 0.001^{***}$											

Table S9: Multiple linear regression analysis of within-occupation skill change compared to 2010 O*NET skill profiles. Data varies by six-digit SOC code from 2011 through 2017 for a total of 479 data points. All variables were centered and standardized before analysis, thus eliminating potentially different units from the technology exposure scores.

10 Predicting Wage Bill Change

Most automation studies compare their exposure scores to occupations' change in employment share, change in wages, or change in wage bill share. We repeat this analysis here with the added benefit of including multiple automation exposure scores and controlling for occupations' skill requirements, state, and year. None of the exposure scores are strong predictors of wage bill share but combine into an ensemble model explains 26.4% of the variation and an additional 15.1% of variation compared to a baseline model controlling for state, year, and occupations' skill requirements (see SI Tables S10 & S11). Breaking this result down, we see that this predictive performance is due to exposure scores' ability to predict employment share (see SI Table S13) but only a small amount due to their ability to predict occupations' annual wages (see Table S12). This poor performance when predicting wages may at first seem to contradict some of the AI studies that introduce these scores (e.g., (*23*) find that LLM exposure is greater for high wage occupations), but our analysis differs by controlling for occupations' skill requirements according to BLS O*NET. Our results show that occupations' skill requirements are a good predictor of occupations' annual wages (see Model 11 in Table S12) and that some of that predictive performance is mediated by exposure to AI (see Model 10 and 12).

Dependent Variable: State Wage Bill Share										
Variable	Model 1	Model 2	Model 3	Model 4	Model 5	Model 6	Model 7	Model 8	Model 9	Model 10
Acemoglu & Autor (*8*) Comp.Use	0.162***									-0.000
Acemoglu & Autor (*8*) R.Cog.	-0.324***									-0.238***
Acemoglu & Autor (*8*) R.Man.	0.010									0.105***
ONET Education %college		0.093***								0.070***
Frey & Osborne (*13*) auto			-0.209***							-0.204***
Arntz et al (*14*) auto2				-0.219***						0.237***
ONET Automation Deg.Auto.					-0.216***					0.000
Brynjolfsson et al (*20*) SML						-0.220***				0.000
Felten et al (*21*) AI2							-0.207***			-0.000
Webb (*22*) AI								-0.000		-0.910***
Webb (*22*) Robot								-0.098***		-0.000
Webb (*22*) Software								-0.184***		-0.075
Eloundou et al (*23*) LLM									-0.124***	0.873***
R^2	0.098	0.009	0.048	0.048	0.047	0.049	0.043	0.080	0.015	0.264
adj. R^2	0.097	0.009	0.048	0.048	0.047	0.049	0.043	0.080	0.015	0.263
$p_{val} < 0.1^{*}, p_{val} < 0.01^{**}, p_{val} < 0.001^{***}$										

Table S10: Linear regression analysis of occupations' technology exposure and wage bill share by occupation, state, and year according to the US BLS. Data varies by major occupation, state, and month from January 2010 through 2020 for a total of 11,760 data points. All variables were centered and standardized before analysis thus eliminating units from the various exposure variables.

Dependent Variable: State Wage Bill Share												
Variable	Model 1	Model 2	Model 3	Model 4	Model 5	Model 6	Model 7	Model 8	Model 9	Model 10	Model 11	Model 12
Acemoglu & Autor (*8*) Comp.Use	0.162***									-0.000		0.000
Acemoglu & Autor (*8*) R.Cog.	-0.324***									-0.238***		-0.197***
Acemoglu & Autor (*8*) R.Man.	0.010									0.105***		0.303***
ONET Education %college		0.093***								0.070***		0.004
Frey & Osborne (*13*) auto			-0.209***							-0.204***		-0.171***
Arntz et al (*14*) auto2				-0.219***						0.237***		0.002
ONET Automation Deg.Auto.					-0.216***					0.000		0.000
Brynjolfsson et al (*20*) SML						-0.220***				0.000		-0.106
Felten et al (*21*) AI2							-0.207***			-0.000		0.000
Webb (*22*) AI								-0.000		-0.910***		-0.000
Webb (*22*) Robot								-0.098***		-0.000		-0.007
Webb (*22*) Software								-0.184***		-0.075		-0.186**
Eloundou et al (*23*) LLM									-0.124***	0.873***		0.379***
O*NET PCA	No	No	No	No	No	No	No	No	No	No	Yes	Yes
Year F.E.	No	No	No	No	No	No	No	No	No	No	Yes	Yes
State F.E.	No	No	No	No	No	No	No	No	No	No	Yes	Yes
R^2	0.098	0.009	0.048	0.048	0.047	0.049	0.043	0.080	0.015	0.264	0.412	0.563
adj. R^2	0.097	0.009	0.048	0.048	0.047	0.049	0.043	0.080	0.015	0.263	0.409	0.560
$p_{val} < 0.1^{*}, p_{val} < 0.01^{**}, p_{val} < 0.001^{***}$												

Table S11: LASSO regression analysis of occupations' technology exposure and wage bill share by occupation, state, and year according to the US BLS. Data varies by major occupation, state, and month from January 2010 through 2020 for a total of 11,760 data points. All variables were centered and standardized before analysis thus eliminating units from the various exposure variables.

Dependent Variable: Log_{10} Avg. Annual Wage												
Variable	Model 1	Model 2	Model 3	Model 4	Model 5	Model 6	Model 7	Model 8	Model 9	Model 10	Model 11	Model 12
Acemoglu & Autor (*8*) Comp.Use	0.377***									-0.000		0.127***
Acemoglu & Autor (*8*) R.Cog.	0.034**									0.105***		-0.000
Acemoglu & Autor (*8*) R.Man.	-0.057***									-0.224***		0.180***
ONET Education %college		0.055***								0.042***		-0.047***
Frey & Osborne (*13*) auto			-0.241***							-0.195***		-0.070***
Arntz et al (*14*) auto2				0.111***						-0.176***		-0.241***
ONET Automation Deg.Auto.					0.188***					-0.097*		0.000
Brynjolfsson et al (*20*) SML						0.155***				-0.582***		-0.653***
Felten et al (*21*) AI2							0.260***			0.408***		0.675***
Webb (*22*) AI								0.000		-0.908***		0.574***
Webb (*22*) Robot								-0.474***		0.000		0.478***
Webb (*22*) Software								0.548***		0.894***		-0.370***
Eloundou et al (*23*) LLM									0.305***	0.569***		0.000
O*NET PCA	No	No	No	No	No	No	No	No	No	No	Yes	Yes
Year F.E.	No	No	No	No	No	No	No	No	No	No	Yes	Yes
State F.E.	No	No	No	No	No	No	No	No	No	No	Yes	Yes
R^2	0.198	0.003	0.061	0.013	0.036	0.026	0.068	0.154	0.093	0.270	0.823	0.901
adj. R^2	0.198	0.003	0.061	0.013	0.036	0.026	0.068	0.154	0.093	0.269	0.822	0.900
$p_{val} < 0.1^{*}, p_{val} < 0.01^{**}, p_{val} < 0.001^{***}$												

Table S12: LASSO regression analysis of occupations' technology exposure and average annual wage by occupation, state, and year according to the US BLS. Data varies by major occupation, state, and month from January 2010 through 2020 for a total of 11,760 data points. All variables were centered and standardized before analysis thus eliminating units from the various exposure variables.

Dependent Variable: Log_{10} Employment Share By Occupation & State												
Variable	Model 1	Model 2	Model 3	Model 4	Model 5	Model 6	Model 7	Model 8	Model 9	Model 10	Model 11	Model 12
Acemoglu & Autor (*8*) Comp.Use	-0.003									-0.300^{***}		-0.089^{**}
Acemoglu & Autor (*8*) R.Cog.	-0.103^{***}									0.003		0.090^{***}
Acemoglu & Autor (*8*) R.Man.	0.145^{***}									0.125^{***}		0.170^{***}
ONET Education %college		-0.068^{***}								-0.000		-0.053^{***}
Frey & Osborne (*13*) auto			-0.225^{***}							-0.273^{***}		-0.191^{***}
Arntz et al (*14*) auto2				-0.324^{***}						0.000		-0.000
ONET Automation Deg.Auto.					-0.322^{***}					0.000		0.000
Brynjolfsson et al (*20*) SML						-0.296^{***}				0.319^{***}		0.000
Felten et al (*21*) AI2							-0.315^{***}			-0.000		-0.000
Webb (*22*) AI								0.622^{***}		-0.141^{*}		-0.000
Webb (*22*) Robot								0.341^{***}		0.000		0.000
Webb (*22*) Software								-1.275^{***}		-0.691^{***}		-0.464^{***}
Eloundou et al (*23*) LLM									-0.260^{***}	0.495^{***}		0.196^{***}
O*NET PCA	No	No	No	No	No	No	No	No	No	No	Yes	Yes
Year F.E.	No	No	No	No	No	No	No	No	No	No	Yes	Yes
State F.E.	No	No	No	No	No	No	No	No	No	No	Yes	Yes
R^2	0.055	0.007	0.058	0.106	0.105	0.092	0.100	0.214	0.068	0.323	0.388	0.596
adj. R^2	0.055	0.007	0.058	0.106	0.105	0.091	0.100	0.214	0.068	0.322	0.384	0.593
$p_{val} < 0.1^{*}, p_{val} < 0.01^{**}, p_{val} < 0.001^{***}$												

Table S13: LASSO regression analysis of occupations' technology exposure and employment share by occupation, state, and year according to the US BLS. Data varies by state, and month from January 2010 through 2020 for a total of 11,760 data points. All variables were centered and standardized before analysis thus eliminating units from the various exposure variables.

References

1. David H Autor, Lawrence F Katz, and Melissa S Kearney. Trends in US wage inequality: Revising the revisionists. *The Review of Economics and Statistics*, 90(2):300–323, 2008.

2. Daron Acemoglu. Technical change, inequality, and the labor market. *Journal of economic literature*, 40(1):7–72, 2002.

3. Matthew Hutson. Self-taught artificial intelligence beats doctors at predicting heart attacks. *Science*, 80, 2017.

4. Erik R Ranschaert, André J Duerinckx, Paul Algra, Elmar Kotter, Hans Kortman, and Sergey Morozov. Advantages, challenges, and risks of artificial intelligence for radiologists. In *Artificial Intelligence in Medical Imaging*, pages 329–346. Springer, 2019.

5. Bernard F King. Artificial intelligence and radiology: what will the future hold? *Journal of the American College of Radiology*, 15(3):501–503, 2018.

6. Robert Schier. Artificial intelligence and the practice of radiology: an alternative view. *Journal of the American College of Radiology*, 15(7):1004–1007, 2018.

7. Nikhil Naik, Scott Duke Kominers, Ramesh Raskar, Edward L Glaeser, and César A Hidalgo. Computer vision uncovers predictors of physical urban change. *Proceedings of the National Academy of Sciences*, 114(29):7571–7576, 2017.

8. Daron Acemoglu and David Autor. Skills, tasks and technologies: Implications for employment and earnings. In *Handbook of labor economics*, volume 4, pages 1043–1171. Elsevier, 2011.

9. Daron Acemoglu and Pascual Restrepo. Robots and jobs: Evidence from US labor markets. *Journal of Political Economy*, 128(6):2188–2244, 2020.

10. Morgan R Frank, David Autor, James E Bessen, Erik Brynjolfsson, Manuel Cebrian, David J Deming, Maryann Feldman, Matthew Groh, José Lobo, Esteban Moro, et al. Toward understanding the impact of artificial intelligence on labor. *Proceedings of the National Academy of Sciences*, 116(14):6531–6539, 2019.

11. Ahmad Alabdulkareem*, Morgan R. Frank*, Lijun Sun, Bedoor AlShebli, César Hidalgo, and Iyad Rahwan. Unpacking the polarization of workplace skills. *Science Advances*, 4(7), 2018. (*authors contributed equally).

12. Michael J Handel. The O*NET content model: strengths and limitations. *Journal for Labour Market Research*, 49(2):157–176, 2016.

13. Carl Benedikt Frey and Michael A. Osborne. The future of employment: How susceptible are jobs to computerisation? *Technological Forecasting and Social Change*, 114:254 – 280, 2017.

14. Melanie Arntz, Terry Gregory, and Ulrich Zierahn. The Risk of Automation for Jobs in OECD Countries. Technical report, 2016.

15. Mica Pajarinen, Petri Rouvinen, and Anders Ekeland. Computerization threatens one-third of Finnish and Norwegian employment. *ETLA Brief*, (34), 2015.

16. Carsten Brzeski and Inga Burk. Die Roboter kommen: Folgen der Automatisierung für den deutschen Arbeitsmarkt [The Robots are coming: Consequences of Automation for the German Labor Market]. *INGDiBa Economic Research*, 30, 2015.

17. Jeremy Bowles. The computerisation of european jobs–who will win and who will lose from the impact of new technology onto old areas of employment. *Bruegel blog*, 17, 2014.

18. Morgan R Frank, Lijun Sun, Manuel Cebrian, Hyejin Youn, and Iyad Rahwan. Small cities face greater impact from automation. *Journal of The Royal Society Interface*, 15(139):20170946, 2018.

19. Daron Acemoglu and Pascual Restrepo. Tasks, automation, and the rise in US wage inequality. *Econometrica*, 90(5):1973–2016, 2022.

20. Erik Brynjolfsson, Tom Mitchell, and Daniel Rock. What can machines learn, and what does it mean for occupations and the economy? In *AEA Papers and Proceedings*, volume 108, pages 43–47, 2018.

21. Edward W Felten, Manav Raj, and Robert Seamans. A Method to Link Advances in Artificial Intelligence to Occupational Abilities. In *AEA Papers and Proceedings*, volume 108, pages 54–57, 2018.

22. Michael Webb. The impact of artificial intelligence on the labor market. *Available at SSRN 3482150*, 2019.

23. Tyna Eloundou, Sam Manning, Pamela Mishkin, and Daniel Rock. GPTs are GPTs: Labor market impact potential of LLMs. *Science*, 384(6702):1306–1308, 2024.

24. Christina Gathmann and Uta Schönberg. How general is human capital? A task-based approach. *Journal of Labor Economics*, 28(1):1–49, 2010.
